# Associations of C-reactive protein, triglyceride–glucose index, and the C-reactive protein–triglyceride glucose index with multistate trajectories in the cardiovascular–renal–diabetes cluster

**DOI:** 10.3389/fendo.2026.1758467

**Published:** 2026-03-16

**Authors:** Hui Li, Liuyu Chen, Mengyi Wang, Wenke Cheng, Zhongyan Du, Yuli Huang

**Affiliations:** 1 Department of Cardiology, The First Affiliated Hospital of Bengbu Medical University, Bengbu, China; 2 Zhejiang Key Laboratory of Blood-Stasis-Toxin Syndrome, Zhejiang Chinese Medical University, Hangzhou, China; 3 School of Humanities and Management, Zhejiang Chinese Medical University, Hangzhou, China; 4 School of Basic Medical Sciences, Zhejiang Chinese Medical University, Hangzhou, China; 5 Zhejiang Engineering Research Center for “Preventive Treatment” Smart Health of Traditional Chinese Medicine, Zhejiang Chinese Medical University, Hangzhou, China

**Keywords:** chronic kidney disease, coronary artery disease, C-reactive protein, multimorbidity, triglyceride–glucose index, type 2diabetes mellitus

## Abstract

**Objective:**

To investigate the associations of C-reactive protein (CRP), triglyceride–glucose (TyG) index, and their composite—the CRP–TyG index (CTI)—with sequential trajectories within the cardiovascular–renal–diabetes (CRD) cluster, including incident coronary artery disease (CAD), type 2 diabetes mellitus (T2DM), chronic kidney disease (CKD), and multimorbidity.

**Methods:**

We analyzed 333,698 participants from the UK Biobank who were free of CAD, T2DM, and CKD at baseline. CRP and TyG were assessed individually and jointly through the CTI. Multistate Cox models were applied to evaluate six predefined transitions within the CRD cluster, with multimorbidity defined as the coexistence of at least two of CAD, T2DM, and CKD. Potential nonlinearity was assessed using restricted cubic splines, and time-dependent effects were examined with piecewise analyses. Joint exposure analyses assessed synergistic effects of CRP and TyG, while receiver operating characteristic (ROC) curves compared the predictive performance of CRP, TyG, their combination, and CTI. Subgroup and sensitivity analyses were performed to test heterogeneity and robustness.

**Results:**

During a median follow-up of 15.31 years (IQR, 14.54–16.03 years), CTI was consistently associated with higher risks of CAD (HR per 1-SD: 1.23, 95% CI: 1.21–1.25), T2DM (1.88, 95% CI: 1.84–1.92), CKD (1.22, 95% CI: 1.19–1.25), and multimorbidity (1.59, 95% CI: 1.55–1.64), outperforming CRP and TyG individually. CTI exhibited trajectory-specific heterogeneity, with nonlinear associations observed in most baseline-to-disease transitions (P for nonlinearity <0.05) and predominantly linear associations during progression from single diseases to multimorbidity (all P for nonlinearity >0.05). Moreover, higher CTI was associated with a time-dependent cumulative increase in multimorbidity risk. In joint exposure analyses, participants with both high CRP and high TyG had the greatest risks across outcomes, including CAD (HR 1.48, 95% CI: 1.40–1.56), CKD (HR 1.52, 95% CI: 1.40–1.64), T2DM (HR 3.63, 95% CI: 3.35–3.93), and multimorbidity (HR 2.64, 95% CI: 2.47–2.82).

**Conclusions:**

CRP, TyG, and CTI were strongly associated with both the onset and progression of the cardiovascular–renal–diabetes cluster. By integrating metabolic and inflammatory risk signals, CTI outperformed its individual components, underscoring its clinical utility for refined risk stratification and for guiding early, stage-specific prevention strategies.

## Graphical Abstract

**Figure:**
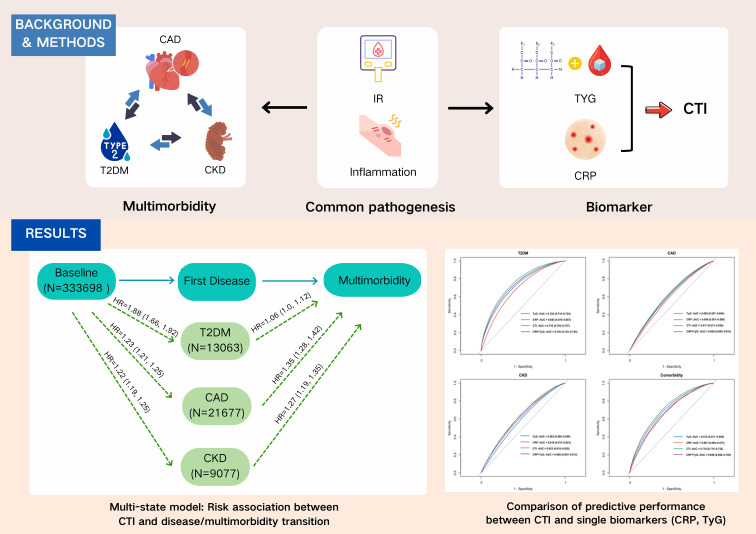
CTI integrates metabolic (TyG) and inflammatory (CRP) signals to capture shared mechanisms within the cardiovascular–renal–diabetes cluster. Multistate analyses depict transitions from baseline to first incident CAD, T2DM, or CKD and subsequent progression to multimorbidity, showing higher CTI-associated risks across trajectories and improved discrimination compared with CRP, TyG, and their combination. CRP, C-reactive protein; TyG, triglyceride–glucose index; CTI, C-reactive protein–triglyceride–glucose index; T2DM, type 2 diabetes mellitus; CAD, coronary artery disease; CKD, chronic kidney disease.

## Highlights

- CTI outperformed CRP, TyG, and their combination in predicting CAD, T2DM, CKD, and multimorbidity.
- CTI-related risks intensified over time, with multimorbidity becoming markedly elevated beyond 5 years.
- CTI provides refined risk stratification and supports stage-specific prevention in the CRD cluster.

## Introduction

Coronary artery disease (CAD), type 2 diabetes mellitus (T2DM), and chronic kidney disease (CKD) are among the most prevalent metabolic disorders worldwide. Ischemic heart disease, the dominant clinical manifestation of coronary artery disease, remains the leading single cause of death worldwide, accounting for approximately 9.1 million deaths in 2021, as reported by the World Health Organization (1). Global Burden of Disease (GBD) estimates further suggest that about 197 million people were living with ischemic heart disease in 2019 (2). Diabetes affected an estimated 589 million adults aged 20–79 years worldwide and is projected to increase substantially; it was associated with approximately 3.4 million deaths in 2024, according to the International Diabetes Federation (3). Chronic kidney disease also represents a major global health burden, affecting more than 800 million people globally, often reported as approximately 850 million in global kidney health reports, and accounting for an estimated 1.48 million deaths in 2023 based on GBD-aligned estimates (4). With population aging and the rising burden of metabolic diseases, their coexistence has emerged as a major public health challenge (5, 6). Epidemiologic data demonstrate substantial overlap: the presence of one condition markedly increases the risk of the others and worsens outcomes. Shared pathophysiologic pathways—metabolic dysfunction and chronic inflammation—interact bidirectionally, reinforcing a vicious cycle of excess morbidity and mortality (6). Although distinct entities, CAD, CKD, and T2DM converge on these mechanisms and mutually accelerate disease progression.

Insulin resistance (IR), the hallmark of T2DM, represents a key metabolic link. IR promotes atherogenesis through dyslipidemia, increasing CAD risk, and activates the renin–angiotensin–aldosterone system, predisposing to CKD. Conversely, myocardial ischemia in CAD exacerbates systemic metabolic dysregulation and IR, while CKD-related uremic toxins impair insulin signaling, further worsening IR (7–9). Chronic inflammation similarly drives cross-disease progression. In T2DM, adipose-derived cytokines (e.g., TNF-α, IL-6) impair β-cell function, aggravate glycemic dysregulation, and promote plaque instability, elevating CAD risk. In CAD, plaque rupture provokes systemic inflammation that accelerates renal fibrosis and CKD progression. In turn, CKD-associated systemic inflammation intensifies coronary plaque inflammation, hastening CAD (9–12). These intersecting mechanisms underpin the marked tendency toward comorbidity. Mapping the dynamic trajectories of the cardiovascular–renal–diabetes (CRD) cluster—from disease-free status to single conditions to multimorbidity—and identifying biomarkers driving these transitions are essential for early prevention and intervention.

The triglyceride–glucose (TyG) index is a well established surrogate for IR, valued for its simplicity and reliance on routine laboratory measures, enabling broad use in large-scale studies (13–15). C-reactive protein (CRP), a sensitive marker of low-grade inflammation, has likewise been associated with CAD, CKD, and T2DM. However, each marker alone has limitations; TyG captures metabolic dysfunction but not inflammation, whereas CRP reflects inflammation but not metabolic abnormalities (13, 16, 17). Recognizing the interplay between IR and inflammation, the CRP–TyG index (CTI) was proposed as a composite marker integrating both pathways. Preliminary evidence indicates that CTI offers superior predictive performance for CAD, CKD, and T2DM compared with TyG or CRP alone (18–21).

Nevertheless, important gaps remain. First, CTI has not been systematically evaluated across disease progression, especially from single disease to multimorbidity. Second, potential heterogeneity in progression trajectories by initial disease is poorly understood. Third, reliance on conventional Cox regression models, which precludes assessment of sequential disease ordering and competing risks, potentially leading to biased risk estimates.

To address these gaps, we applied multistate models in a large prospective cohort to evaluate associations of TyG, CRP, and CTI with transitions within the CRD cluster, aiming to clarify their roles in disease dynamics and to inform risk stratification and stage-specific prevention strategies.

## Method

### Study population

This analysis was conducted within the UK Biobank, a large prospective cohort that recruited 502,128 participants aged 40–69 years between 2006 and 2010. At baseline, sociodemographic characteristics, lifestyle factors, physical measurements, and biological samples were collected using standardized protocols. Health outcomes were identified through linkage to national death registries and hospital episode statistics, with detailed study design reported elsewhere (22).

Participants with prevalent CAD (n=27,620), heart failure (n=828), valvular heart disease (n=2,534), cardiomyopathy (n=381), arrhythmias (n=10,642), stroke (n=5,801), T2DM (n=19,580), or CKD (n=3,535) were excluded. The diagnostic details are provided in **Supplementary Table 1**. We also excluded individuals with pregnancy (n=140), cancer (n=40,301), and those lacking complete biomarker data required for CTI calculation (n=57,068).The final analytic sample comprised 333,698 participants free of cardiovascular disease, diabetes, chronic kidney disease, cancer, or pregnancy at baseline.

The UK Biobank received ethical approval from the North West Multi-Centre Research Ethics Committee (reference 21/NW/0157), and all participants provided written informed consent. This study adhered to the STROBE guidelines for reporting observational research.

### Calculation of the TyG index and CTI

CRP was quantified using an immunoturbidimetric assay on the Beckman Coulter AU5800 platform (Beckman Coulter, UK). Triglycerides and plasma glucose were measured using enzymatic methods on the same analyzer.

The CTI was developed to jointly capture systemic inflammation and insulin resistance, integrating CRP and the TyG index (18, 23). The TyG index was calculated as: TyG=ln(TG [mg/dL] × plasma glucose [mg/dL])/2, where plasma glucose refers to random plasma glucose in the UK Biobank. The CTI was then derived according to the established formula: CTI = 0.412×ln(CRP [mg/L])+TyG.

### Ascertainment of CAD, T2DM, CKD, and mortality

Incident CAD, T2DM, and CKD were identified using the UK Biobank First Occurrences dataset (Category 1712), which integrates data from primary care records, hospital admissions, death registries, and self-reports, mapped to 3-character International Classification of Diseases, Tenth Revision (ICD-10) codes. CAD was defined by ICD-10 codes I20–I25, T2DM by E11, and CKD by N18.

All-cause mortality was determined through linkage to national death registries, including NHS Digital (England and Wales) and the NHS Central Register (Scotland), with dates of death obtained from official death certificates.

Outcome data were available through August 1, 2024, for CAD and T2DM; July 1, 2024, for CKD; and July 8, 2024, for mortality. To ensure consistency across outcomes, July 1, 2024, was chosen as the common study end date. Participants were followed from the date of enrollment until the occurrence of multimorbidity, death, loss to follow-up, or July 1, 2024, whichever occurred first.

### Primary outcome and transition pathways

**Figure 1:**
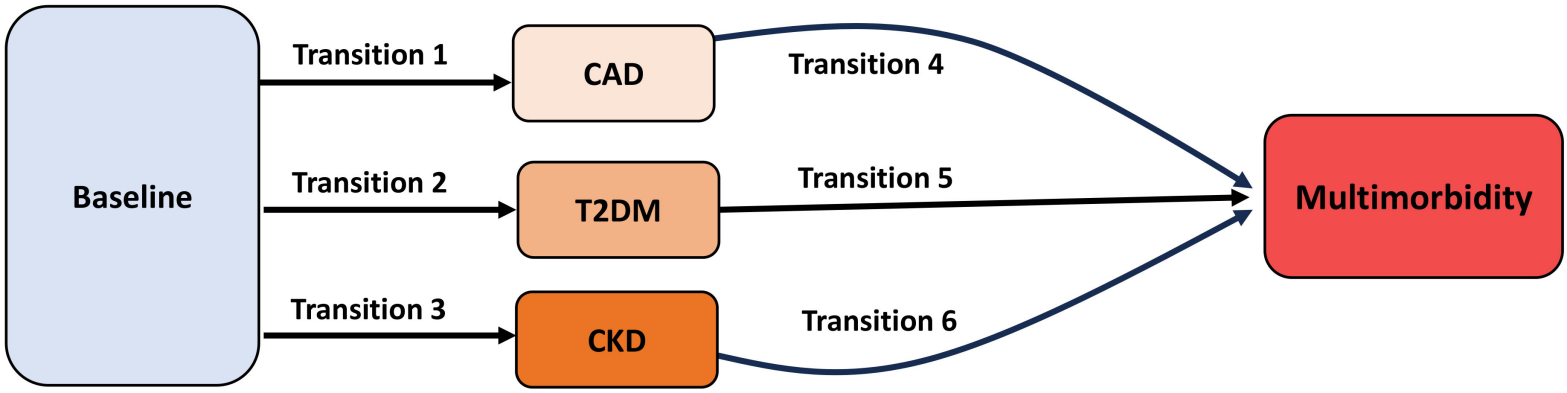
Transition pathways in the cardiovascular–renal–diabetes (CRD) cluster. Six predefined transitions were modeled: baseline to CAD (transition 1), baseline to T2DM (transition 2), baseline to CKD (transition 3), CAD to multimorbidity (transition 4), T2DM to multimorbidity (transition 5), and CKD to multimorbidity (transition 6). Multimorbidity was defined as the coexistence of at least two conditions among CAD, T2DM, and CKD.

The primary outcome was the development of multimorbidity, defined as the occurrence of at least two of the following conditions: CAD, T2DM, and CKD. Disease progression was evaluated through six transition pathways: (1) baseline to incident CAD, (2) baseline to incident T2DM, (3) baseline to incident CKD, (4) CAD to multimorbidity, (5) T2DM to multimorbidity, and (6) CKD to multimorbidity (**Figure 1**).

### Assessment of covariates

Baseline characteristics were obtained from standardized touchscreen questionnaires, nurse-led interviews, physical measurements, and biochemical assays. Demographic factors included age, sex, and self-reported race (White vs. non-White). Socioeconomic status was assessed using the Townsend Deprivation Index (TDI, a validated area-based measure of material deprivation), education level (college degree or equivalent vs. high school or below), and average household income (<£30,999 or ≥£30,999). Lifestyle factors comprised physical activity (classified as low, moderate, or high according to the International Physical Activity Questionnaire), smoking status (active or non-active), alcohol consumption (active or non-active). Furthermore, a cumulative dietary risk score was constructed from the baseline touch-screen questionnaire to characterize individual-level dietary patterns. This composite score summarized an overall adverse dietary pattern, capturing higher intake of high-fat and saturated-fat–rich foods such as processed and red meat, full-cream milk, and spreads, as well as lower intake of protective foods such as fruits, vegetables, and fish. It also incorporated other diet-related behaviors, including cereal, salt, and water intake. Each adverse dietary component contributed one point to the cumulative score, resulting in a scale from 0 (most favorable) to 9 (least favorable). The score was classified as low (≤6 points) or high (≥7 points), with details provided in **Supplementary Table 2** (24). Clinical variables included body mass index (BMI), fasting duration (hours since last meal), glycated hemoglobin (HbA1c), hypertension, and use of lipid-lowering or antihypertensive agents. Biochemical analytes were quantified with standardized enzymatic assays on the Beckman Coulter AU5800 platform. For all categorical variables, responses recorded as “prefer not to answer,” “do not know,” or “missing” were classified as a separate category.

To identify potential confounders of the associations between CTI and outcomes, we constructed a directed acyclic graph (DAG) using Dagitty (www.dagitty.net), in line with contemporary causal inference practices (25). The DAG identified a minimally sufficient adjustment set comprising age, sex, BMI, race, physical activity, hypertension, cumulative dietary risk score, smoking status, alcohol consumption, lipid-lowering therapy, HbA1c, and fasting hours (**Supplementary Figure 1**). Cholesterol levels and antihypertensive therapy were regarded as potential mediators rather than confounders: cholesterol reflects downstream metabolic alterations along the pathway from CTI-related dysmetabolism to clinical outcomes, whereas antihypertensive therapy represents a treatment response to elevated blood pressure. To avoid overadjustment bias and preserve estimates of the total effect of CTI, these variables were excluded from the final models.

In addition, covariates were evaluated empirically and retained if they satisfied at least one of two criteria for any outcome: (1) inducing >10% change in the β coefficient for the CTI–outcome association, or (2) demonstrating an independent association with the outcome at P < 0.1 (26, 27). To ensure consistency and minimize residual confounding, all covariates meeting either criterion were included in the final models (**Supplementary Table 3**).

### Statistical analysis

Missing data for continuous variables were imputed using mean values, and categorical variables with missing responses (e.g., “prefer not to answer”) were grouped into a separate category. Continuous variables are reported as means with standard deviations (SDs), and categorical variables as counts and percentages. Exposures of interest included CRP, the TyG index, and CTI. To ensure comparability across measurement scales, all exposures were standardized as Z-scores (mean = 0, SD = 1). Associations of CRP, TyG, and CTI with incident CAD, T2DM, CKD, and multimorbidity were initially evaluated using conventional Cox proportional hazards models, with hazard ratios (HRs) and 95% confidence intervals (CIs) estimated per 1-SD increase in standardized exposures. Disease trajectories were analyzed using multistate Cox models with a clock-forward approach (mstate package), which extend conventional Cox regression by modeling sequential transitions and competing risks. Six pathways were prespecified: (1) baseline to incident CAD, (2) baseline to incident T2DM, (3) baseline to CKD, (4) CAD to multimorbidity, (5) T2DM to multimorbidity, and (6) CKD to multimorbidity. For participants who developed multiple disease states on the same date, temporal ordering could not be determined; therefore, they were excluded from the multistate models. However, these individuals remained included in the conventional Cox regression analyses.

Time-varying effects for transitions from CAD, T2DM, or CKD to multimorbidity were assessed using piecewise Cox models. Interaction terms between exposures (per 1-SD increment) and predefined follow-up intervals (0–<1, 1–<3, 3–<5, and ≥5 years) were included. Follow-up time was split with the survSplit function, and interval-specific HRs with 95% CIs were estimated and displayed in forest plots.

Because CTI was derived from CRP and TyG, receiver operating characteristic (ROC) analyses compared the predictive performance of CTI, CRP, TyG, and the combination of CRP and TyG for CRD cluster. Discrimination was quantified using the area under the curve (AUC), indicating each marker’s ability to distinguish between individuals with and without the outcome.

The TyG index was dichotomized at the cohort median (8.65) into low (<8.65) and high (≥8.65) categories. CRP was dichotomized at 5 mg/L to define chronic inflammation status, with low (<5 mg/L) and high (≥5 mg/L) groups, both coded as binary variables. Joint exposure was defined by cross-classifying these categories, yielding four groups with low CRP/low TyG as the reference. Relative risks for multimorbidity were compared across groups. Additive interaction was assessed using the relative excess risk due to interaction (RERI), attributable proportion (AP), and synergy index (S), with 95% CIs estimated via the delta method. Multiplicative interaction was tested using product terms and likelihood ratio tests. Synergy was considered present if either additive or multiplicative interaction was significant.

To investigate potential non-linear dose–response relationships between CTI and transitions within the CRD cluster, multi-state Cox models were applied for six predefined pathways. Non-linearity was assessed using restricted cubic splines (RCS), with knot numbers (3–6) selected by minimizing the Akaike Information Criterion (AIC). For each pathway, likelihood ratio tests compared spline and linear models to evaluate evidence of non-linearity. To mitigate outlier influence, CTI values were winsorized at the 1st and 99th percentiles within transition-specific risk sets. This transition-specific approach robustly captured potential non-linear effects of CTI on CRD cluster onset and progression across pathways.

Effect modification was examined by age (≤55 vs. >55 years), sex, BMI (<30 vs. ≥30 kg/m²), dietary risk score (low vs. high), hypertension (yes vs. no), and physical activity (low, moderate, high). Interactions were tested using likelihood ratio comparisons, and stratum-specific HRs with 95% CIs were reported per 1-SD increment.

Sensitivity analyses included: (1) excluding incident CAD, T2DM, or CKD cases occurring within two years of enrollment to minimize reverse causation; (2) multiple imputation of missing covariates with results pooled by Rubin’s rules; (3) restricting exposures to the 2.5th–97.5th percentiles; (4) complete-case analysis excluding participants with missing baseline data; (5) additional adjustment for low-density lipoprotein cholesterol and antihypertensive therapy; and (6) inclusion of a direct baseline-to-multimorbidity transition in the multistate framework.

All analyses were conducted in R version 4.2.3. Two-sided P values <0.05 were considered statistically significant.

## Results

A total of 333,698 participants were included, of whom 1,306 developed CAD, T2DM, or CKD on the same day, making temporal ordering indeterminate. Sample sizes for the six transition pathways were baseline to T2DM (n=13,063), baseline to CAD (n=21,677), baseline to CKD (n=9,077), T2DM to multimorbidity (n=1,685), CAD to multimorbidity (n=2,265), and CKD to multimorbidity (n=1,094). Compared with the overall cohort (mean age 55.6 years; 44.9% men), participants across all transition pathways were older, with a higher proportion of men. They exhibited adverse cardiometabolic profiles, including higher body mass index, HbA1c, CRP, CTI, and TyG levels, alongside lower HDL cholesterol. Hypertension was more common, with greater use of antihypertensive and lipid-lowering therapies.

Sociodemographic and lifestyle differences were also evident. Participants in transition pathways had higher Townsend deprivation index scores, lower household income, and lower educational attainment. They more frequently reported high dietary risk, lower physical activity, and active smoking, while non-drinking status was more common than in the overall cohort. Detailed baseline characteristics for the total population and transition-specific subgroups are shown in **Table 1**.

**Table 1:** Baseline characteristics of the 333,698 individuals grouped by six transition stages.

Variable	Total	Baseline to T2DM	Baseline to CAD	Baseline to CKD	T2DM to comorbidity	CAD to comorbidity	CKD to comorbidity
**Sample size**	**333698**	**13063**	**21677**	**9077**	**1685**	**2265**	**1094**
Age (years)	55.6 (8.09)	57.53 (7.68)	59.33 (7.11)	61.35 (6.43)	59.84 (6.90)	60.73 (6.67)	62.00 (5.97)
White (%)	314538 (94.3%)	12790 (87.5%)	22401 (94.9%)	10529 (94.8%)	2885 (89.3%)	3871 (92.3%)	2905 (93.1%)
BMI kg/m^2^	27.12 (4.58)	31.19 (5.56)	28.16 (4.62)	28.68 (4.97)	31.18 (5.60)	29.94 (5.30)	29.75 (5.30)
Men (%)	149904 (44.9%)	8006 (54.8%)	14937 (63.3%)	4888 (44.0%)	1933 (59.8%)	2589 (61.7%)	1728 (55.4%)
TDI	-1.38 (3.05)	-0.54 (3.42)	-1.17 (3.16)	-1.20 (3.12)	-0.44 (3.38)	-0.73 (3.32)	-0.82 (3.30)
Fasting hours	3.80 (2.45)	4.07 (2.77)	3.90 (2.55)	3.91 (2.46)	4.08 (2.74)	4.05 (2.74)	4.03 (2.69)
HDL (mmol/L)	1.47 (0.38)	1.26 (0.32)	1.36 (0.36)	1.41 (0.37)	1.24 (0.31)	1.29 (0.34)	1.32 (0.35)
LDL (mmol/L)	3.65 (0.83)	3.67 (0.88)	3.79 (0.88)	3.64 (0.88)	3.69 (0.90)	3.71 (0.89)	3.63 (0.90)
TC (mmol/L)	5.82 (1.08)	5.76 (1.17)	5.92 (1.14)	5.78 (1.15)	5.77 (1.18)	5.80 (1.16)	5.71 (1.19)
HbA1c (mmol/mol)	35.03 (4.42)	41.50 (9.65)	35.95 (4.98)	36.33 (5.48)	41.49 (9.46)	38.93 (7.68)	38.26 (8.11)
CRP (mg/L)	2.48 (4.14)	4.23 (5.37)	3.14 (4.92)	3.66 (5.43)	4.61 (5.80)	4.25 (5.72)	4.39 (6.05)
CTI	8.8 (0.78)	9.51 (0.76)	9.08 (0.74)	9.11 (0.74)	9.55 (0.74)	9.38 (0.74)	9.33 (0.74)
TyG	8.68 (0.55)	9.12 (0.60)	8.85 (0.54)	8.82 (0.53)	9.13 (0.57)	9.01 (0.56)	8.95 (0.55)
Hypertension (%)	74033 (22.2%)	6222 (42.6%)	8542 (36.2%)	5037 (45.4%)	1644 (50.9%)	2073 (49.4%)	1660 (53.2%)
Average household_income,£							
< 30999	128865 (38.6%)	7280 (49.8%)	11128 (47.1%)	5878 (52.9%)	1745 (54.0%)	2279 (54.4%)	1792 (57.5%)
≥30999	158487 (47.5%)	4835 (33.1%)	8704 (36.9%)	3062 (27.6%)	858 (26.6%)	1108 (26.4%)	721 (23.1%)
Unkown	46346 (13.9%)	2495 (17.1%)	3773 (16.0%)	2162 (19.5%)	628 (19.4%)	806 (19.2%)	606 (19.4%)
Cumulative dietary risk							
Low risk	261911 (78.5%)	10341 (70.8%)	17469 (74.0%)	8601 (77.5%)	2272 (70.3%)	3027 (72.2%)	2288 (73.4%)
High risk	59919 (18.0%)	3294 (22.5%)	5000 (21.2%)	1976 (17.8%)	731 (22.6%)	908 (21.7%)	649 (20.8%)
Unknown	11868 (3.6%)	975 (6.7%)	1136 (4.8%)	525 (4.7%)	228 (7.1%)	258 (6.2%)	182 (5.8%)
Physical activity							
Low	46299 (13.9%)	2563 (17.5%)	3403 (14.4%)	1608 (14.5%)	551 (17.1%)	688 (16.4%)	479 (15.4%)
Moderate	105895 (31.7%)	4086 (28.0%)	6950 (29.4%)	3275 (29.5%)	875 (27.1%)	1149 (27.4%)	854 (27.4%)
Hingh	108297 (32.5%)	3793 (26.0%)	7518 (31.8%)	3101 (27.9%)	793 (24.5%)	1125 (26.8%)	799 (25.6%)
Unkown	73207 (21.9%)	4168 (28.5%)	5734 (24.3%)	3118 (28.1%)	1012 (31.3%)	1231 (29.4%)	987 (31.6%)
Education							
College Degree or Equivalent	113150 (33.9%)	3306 (22.6%)	6202 (26.3%)	2313 (20.8%)	605 (18.7%)	820 (19.6%)	539 (17.3%)
High-School or Below	216852 (65.0%)	10992 (75.2%)	17044 (72.2%)	8626 (77.7%)	2535 (78.5%)	3279 (78.2%)	2518 (80.7%)
Unknown	3696 (1.1%)	312 (2.1%)	359 (1.5%)	163 (1.5%)	91 (2.8%)	94 (2.2%)	62 (2.0%)
Lowering lipid drugs							
No	300358 (90.0%)	11411 (78.1%)	19327 (81.9%)	8673 (78.1%)	2354 (72.9%)	3118 (74.4%)	2272 (72.8%)
Yes	32990 (9.9%)	3167 (21.7%)	4246 (18.0%)	2410 (21.7%)	867 (26.8%)	1065 (25.4%)	841 (27.0%)
Missing	350 (0.1%)	32 (0.2%)	32 (0.1%)	19 (0.2%)	10 (0.3%)	10 (0.2%)	6 (0.2%)
Antihypertensives							
No	284351 (85.2%)	10016 (68.6%)	17578 (74.5%)	7060 (63.6%)	1977 (61.2%)	2592 (61.8%)	1778 (57.0%)
Yes	48997 (14.7%)	4562 (31.2%)	5995 (25.4%)	4023 (36.2%)	1244 (38.5%)	1591 (37.9%)	1335 (42.8%)
Missing	350 (0.1%)	32 (0.2%)	32 (0.1%)	19 (0.2%)	10 (0.3%)	10 (0.2%)	6 (0.2%)
Smoking							
Active	34971 (10.5%)	2202 (15.1%)	3676 (15.6%)	1243 (11.2%)	561 (17.4%)	736 (17.6%)	472 (15.1%)
Non-active	297210 (89.1%)	12300 (84.2%)	19771 (83.8%)	9784 (88.1%)	2642 (81.8%)	3419 (81.5%)	2618 (83.9%)
Missing	1517 (0.5%)	108 (0.7%)	158 (0.7%)	75 (0.7%)	28 (0.9%)	38 (0.9%)	29 (0.9%)
Alcohol consumption							
Active	308884 (92.6%)	12689 (86.9%)	21472 (91.0%)	9891 (89.1%)	2751 (85.1%)	3668 (87.5%)	2718 (87.1%)
Non-active	24033 (7.2%)	1847 (12.6%)	2058 (8.7%)	1170 (10.5%)	463 (14.3%)	509 (12.1%)	391 (12.5%)
Missing	781 (0.2%)	74 (0.5%)	75 (0.3%)	41 (0.4%)	17 (0.5%)	16 (0.4%)	10 (0.3%)

TDI, Townsend Deprivation Index; HDL, High-density lipoprotein cholesterol; LDL, Low-density lipoprotein cholesterol; TC, Total cholesterol; HbA1c, Glycated hemoglobin; CRP, C-reactive protein; CTI, C-reactive protein-triglyceride-glucose index; TyG,Triglyceride-glucose index.

Six predefined transitions were modeled: baseline to CAD (transition 1), baseline to T2DM (transition 2), baseline to CKD (transition 3), CAD to multimorbidity (transition 4), T2DM to multimorbidity (transition 5), and CKD to multimorbidity (transition 6).

### Conventional cox and multistate models

In conventional Cox proportional hazards models, each 1-SD increment in CRP, TyG, and CTI was associated with significantly higher risks of T2DM, CAD, CKD, and multimorbidity (all P < 0.001; **Table 2**), exhibiting distinct risk gradients. For T2DM, the HRs (95% CI) were 1.09 (1.08–1.11) for CRP, 1.67 (1.64–1.69) for TyG, and 1.86 (1.82–1.89) for CTI (strongest association). For CAD, the corresponding HRs were 1.07 (1.06–1.08) for CRP, 1.14 (1.13–1.16) for TyG, and 1.23 (1.21–1.25) for CTI. For CKD, the HRs were similar for CRP (1.10 [1.08–1.11]) and TyG (1.10 [1.08–1.12]), but stronger for CTI (1.23 [1.21–1.26]). For multimorbidity, the HRs were 1.12 (1.11–1.14) for CRP, 1.39 (1.36–1.43) for TyG, and 1.59 (1.55–1.64) for CTI.

**Table 2:** Associations of CRP, TyG, and CTI with dynamic transitions within the cardiovascular–renal–diabetes cluster, as evaluated by conventional Cox regression and multistate models.

	Case	Proportion (%)	HR (95% CI)	*P*-value	HR (95% CI)	*P*-value	HR (95% CI)	*P*-value
Traditional cox model								
*Per 1-SD increase*			**CRP**		**TyG**		**CTI**	
T2DM	15608	4.7%	1.09 (1.08, 1.11)	<0.001	1.67 (1.64, 1.69)	<0.001	1.86 (1.82, 1.89)	<0.001
CAD	24676	7.4%	1.07 (1.06, 1.08)	<0.001	1.14 (1.13, 1.16)	<0.001	1.23 (1.21, 1.25)	<0.001
CKD	11763	3.5%	1.10 (1.08, 1.11)	<0.001	1.10 (1.08, 1.12)	<0.001	1.23 (1.21, 1.26)	<0.001
Comorbidity	6350	1.9%	1.12 (1.11, 1.4)	<0.001	1.39 (1.36, 1.43)	<0.001	1.59 (1.55, 1.64)	<0.001
Multi-state model								
*Per 1-SD increase*			**CRP**		**TyG**		**CTI**	
Baseline → T2DM	13063	3.93%	1.10 (1.08, 1.11)	<0.001	1.69 (1.66, 1.72)	<0.001	1.88 (1.66, 1.92)	<0.001
Baseline → CAD	21677	6.52%	1.07 (1.06, 1.08)	<0.001	1.14 (1.12, 1.16)	<0.001	1.23 (1.21, 1.25)	<0.001
Baseline → CKD	9077	2.73%	1.1 (1.08, 1.11)	<0.001	1.08 (1.06, 1.11)	<0.001	1.22 (1.19, 1.25)	<0.001
T2DM → Comorbidity	1685	13%	1.06 (1.02,1.09)	<0.001	1.0 (0.95, 1.05)	0.902	1.06 (1.0, 1.12)	0.057
CAD → Comorbidity	2265	11.32%	1.08 (1.05, 1.11)	<0.001	1.22 (1.16, 1.27)	<0.001	1.35 (1.28, 1.42)	<0.001
CKD → Comorbidity	1094	12.17%	1.08 (1.04, 1.12)	<0.001	1.17 (1.09, 1.24)	<0.001	1.27 (1.19, 1.37)	<0.001

CRP, C-reactive protein; TyG, triglyceride–glucose index; CTI, C-reactive protein–triglyceride–glucose index; T2DM, type 2 diabetes mellitus; CAD, coronary artery disease; CKD, chronic kidney disease. Traditional Cox models estimated the overall associations of a 1-SD increase (z-standardized) in CRP, TyG, and CTI with the incidence of T2DM, CAD, CKD, and comorbidity. .

Multi-state models were then used to evaluate transition-specific hazards from baseline to each disease state

All models were adjusted for age, sex, body mass index , race, physical activity, hypertension, cumulative dietary-risk score, smoking status, alcohol consumption, lipid-lowering drugs, HbA1c, and fasting hours.

In multistate models, each 1-SD increment in CRP, TyG, and CTI demonstrated transition-specific effects (**Table 2**). For transitions from baseline to single diseases: T2DM (CRP: 1.10 [1.08–1.11]; TyG: 1.69 [1.66–1.72]; CTI: 1.88 [1.84–1.92]); CAD (CRP: 1.07 [1.06–1.08]; TyG: 1.14 [1.12–1.16]; CTI: 1.23 [1.21–1.25]); CKD (CRP: 1.10 [1.08–1.11]; TyG: 1.08 [1.06–1.11]; CTI: 1.22 [1.19–1.25]). For progression from single diseases to multimorbidity: post-T2DM (CRP: 1.06 [1.02–1.09], P < 0.001; TyG: 1.00 [0.95–1.05], P = 0.902; CTI: 1.06 [1.00–1.12], P = 0.057); post-CAD (CRP: 1.08 [1.05–1.11]; TyG: 1.22 [1.16–1.27]; CTI: 1.35 [1.28–1.42]); post-CKD (CRP: 1.08 [1.04–1.12]; TyG: 1.17 [1.09–1.24]; CTI: 1.27 [1.19–1.37]).

### Joint effects of CRP and TyG on risks across the CRD cluster

Using low CRP and low TyG levels as the reference, joint associations were evident in both conventional Cox and multistate models (**Table 3**). In Cox models, compared with the reference, participants with high TyG alone (HR, 2.42; 95% CI, 2.32–2.53), high CRP alone (HR, 1.60; 95% CI, 1.47–1.74), or the joint high-exposure group (HR, 3.55; 95% CI, 3.35–3.75) had substantially higher risks of T2DM (all P < 0.001). Comparable patterns were observed for CAD, CKD, and multimorbidity, with the joint high-exposure group consistently demonstrating the strongest associations: CAD (HR, 1.51; 95% CI, 1.44–1.58), CKD (HR, 1.57; 95% CI, 1.47–1.68), and multimorbidity (HR, 2.64; 95% CI, 2.42–2.88).

**Table 3:** Joint associations of TyG and CRP with dynamic transitions within the cardiovascular–renal–diabetes cluster, assessed using conventional Cox regression and multi-state models.

CRP	Low			High			
TyG	Low exposure	High exposure		Low exposure		High exposure	
Traditional Cox model		HR (95% CI)	*P* value	HR (95% CI)	*P* value	HR (95% CI)	*P* value
T2DM	1 (ref.)	2.42 (2.32, 2.53)	<0.001	1.60 (1.47, 1.74)	<0.001	3.55 (3.35, 3.75)	<0.001
CAD	1 (ref.)	1.25 (1.22, 1.29)	<0.001	1.27 (1.19, 1.36)	<0.001	1.51 (1.44, 1.58)	<0.001
CKD	1 (ref.)	1.13 (1.08, 1.18)	<0.001	1.34 (1.23, 1.45)	<0.001	1.57 (1.47, 1.68)	<0.001
Multimorbidity		1.69 (1.58, 1.80)	<0.001	1.62 (1.43, 1.84)	<0.001	2.64 (2.42, 2.88)	<0.001
Multi-state model							
Baseline → T2DM	1 (ref.)	2.47 (2.28, 2.68)	<0.001	1.61 (1.44, 1.80)	<0.001	3.63 (3.35, 3.93)	<0.001
Baseline → CAD	1 (ref.)	1.24 (1.20, 1.28)	0.087	1.25 (1.17, 1.34)	<0.001	1.48 (1.40, 1.56)	<0.001
Baseline → CKD	1 (ref.)	1.11 (1.06, 1.16)	<0.001	1.34 (1.22, 1.47)	<0.001	1.52 (1.40, 1.64)	<0.001
T2DM → Multimorbidity	1 (ref.)	1.04 (0.89, 1.21)	0.615	1.29 (1.0, 1.66)	0.054	1.21 (1.01, 1.45)	0.034
CAD → Multimorbidity	1 (ref.)	1.30 (1.71, 1.44)	<0.001	1.36 (1.11, 1.67)	0.003	1.84 (1.59, 2.13)	<0.001
CKD → Multimorbidity	1 (ref.)	1.31 (1.12, 1.53)	0.001	1.38 (1.04, 1.83)	0.024	1.65 (1.34, 2.03)	<0.001

T2DM, type 2 diabetes mellitus; CAD, coronary artery disease; CKD, chronic kidney disease; TyG, triglyceride–glucose index; CRP, C-reactive protein;

Reference group: TyG & CRP (both low level), serving as the lowest-risk category.

The TyG index was dichotomized at the cohort median of 8.65 into low (<8.65) and high (≥8.65) groups, creating the binary variable TyG (0=low, 1=high). CRP was dichotomized at 5 mg/L to reflect chronic inflammation status, with low (<5 mg/L) vs high (≥5 mg/L) groups, yielding binary variable CRP (0=low, 1=high).

All models were adjusted for age, sex, body mass index , race, physical activity, hypertension, cumulative dietary-risk score, smoking status, alcohol consumption, lipid-lowering drugs, HbA1c, and fasting hours.

In multistate models, results were consistent across transitions. From baseline to T2DM, HRs were 2.47 (95% CI, 2.28–2.68) for high TyG alone, 1.61 (95% CI, 1.44–1.80) for high CRP alone, and 3.63 (95% CI, 3.35–3.93) for the joint high-exposure group (all P < 0.001). For baseline to CAD, corresponding HRs were 1.24 (95% CI, 1.20–1.28), 1.25 (95% CI, 1.17–1.34), and 1.48 (95% CI, 1.40–1.56); for baseline to CKD, 1.11 (95% CI, 1.06–1.16), 1.34 (95% CI, 1.22–1.47), and 1.52 (95% CI, 1.40–1.64), respectively. In transitions from single diseases to multimorbidity, the joint high-exposure group again showed the highest risks: post-T2DM (HR, 1.21; 95% CI, 1.01–1.45), post-CAD (HR, 1.84; 95% CI, 1.59–2.13), and post-CKD (HR, 1.65; 95% CI, 1.34–2.03).

Interaction analyses further examined whether the combined effects of CRP and TyG exceeded the sum or product of their individual associations (**Table 4**). On the additive scale, significant positive interactions were detected for T2DM (RERI, 0.531 [95% CI, 0.349–0.714]; AP, 0.150 [95% CI, 0.101–0.198]; S, 1.26 [95% CI, 1.16–1.37]; all P < 0.001) and multimorbidity (RERI, 0.326 [95% CI, 0.082–0.57]; AP, 0.124 [95% CI, 0.035–0.213]; S, 1.25 [95% CI, 1.029–1.468]; all P ≤ 0.009), but not for CAD or CKD. In multistate models, additive interactions persisted for baseline to T2DM (RERI, 0.543 [95% CI, 0.341–0.746]; AP, 0.150 [95% CI, 0.097–0.202]; S, 1.26 [95% CI, 1.148–1.374]; all P < 0.001), but not for baseline to CAD, CKD, or transitions from T2DM, CAD, or CKD to multimorbidity. On the multiplicative scale, no significant interactions were observed for any outcome or transition (all P > 0.05).

**Table 4:** Additive and multiplicative interactions between CRP and TyG on risks of dynamic transitions of the cardiovascular–renal–diabetes cluster.

	Additive interactive					Multiplicative interactive	
	Measure	Estimate	Lower	Upper	P-value	HR (95%CI)	P-value
Traditional Cox model							
T2DM	RERI	0.531	0.349	0.714	<0.001	0.918 (0.837, 1.007)	0.071
	AP	0.15	0.101	0.198	<0.001		
	S	1.26	1.157	1.369	<0.001		
CAD	RERI	-0.015	-0.115	0.085	0.77	0.948 (0.879, 1.022)	0.162
	AP	0.01	-0.076	0.056	0.77		
	S	0.972	0.785	1.158	<0.001		
CKD	RERI	0.108	-0.027	0.244	0.117	1.04 (0.944, 1.154)	0.406
	AP	0.069	-0.016	0.154	0.11		
	S	1.23	0.889	1.579	<0.001		
Multimorbidity	RERI	0.326	0.082	0.57	0.009	0.962 (0.838, 1.105)	0.583
	AP	0.124	0.035	0.213	0.006		
	S	1.25	1.029	1.468	<0.001		
Multistate model							
Baseline → T2DM	RERI	0.543	0.341	0.746	<0.001	0.91 (0.823, 1.007)	0.068
	AP	0.150	0.097	0.202	<0.001		
	S	1.261	1.148	1.374	<0.001		
Baseline → CAD	RERI	-0.012	-0.119	0.095	0.83	0.954 (0.879, 1.035)	0.259
	AP	0.008	-0.080	0.065	0.83		
	S	0.976	0.761	1.191	<0.001		
Baseline → CKD	RERI	0.070	-0.085	0.224	0.38	1.022 (0.911, 1.147)	0.709
	AP	0.046	-0.055	0.146	0.37		
	S	1.156	0.77	1.541	<0.001		
T2DM →Multimorbidity	RERI	-0.113	-0.457	0.230	0.52	0.907 (0.686, 1.199)	0.493
	AP	-0.093	-0.375	0.188	0.52		
	S	0.654	-0.041	1.348	0.07		
CAD → Multimorbidity	RERI	0.174	-0.151	0.50	0.29	1.037 (0.824, 1.306)	0.757
	AP	0.095	-0.078	0.268	0.28		
	S	1.263	0.667	1.859	<0.001		
CKD → Multimorbidity	RERI	-0.039	-0.477	0.399	0.86	0.913 (0.667, 1.251)	0.573
	AP	-0.024	-0.29	0.243	0.86		
	S	0.943	0.337	1.550	<0.001		

T2DM, type 2 diabetes mellitus; CAD, coronary artery disease; CKD, chronic kidney disease; TyG, triglyceride–glucose index; CRP, C-reactive protein;.

The TyG index was dichotomized at the cohort median of 8.65 into low (<8.65) and high (≥8.65) groups, creating the binary variable TyG (low vs. high). CRP was dichotomized at 5 mg/L to reflect chronic inflammation status, with low (<5 mg/L) vs high (≥5 mg/L) groups, yielding binary variable CRP (low vs. high).

RERI, relative excess risk due to interaction; AP, attributable proportion due to interaction; S, synergy index; HR, hazard ratio; CI,confidence interval;

Additive interaction models were adjusted for age, sex, body mass index , race, physical activity, hypertension, cumulative dietary-risk score, smoking status, alcohol consumption, lipid-lowering drugs, HbA1c, and fasting hours.

Multiplicative interaction models were adjusted for the same set of covariates as the additiv models, with an additional product term included to capture the interaction between the CRP and TyG.

### Predictive performance of CRP, TyG, and CTI

**Figure 2:**
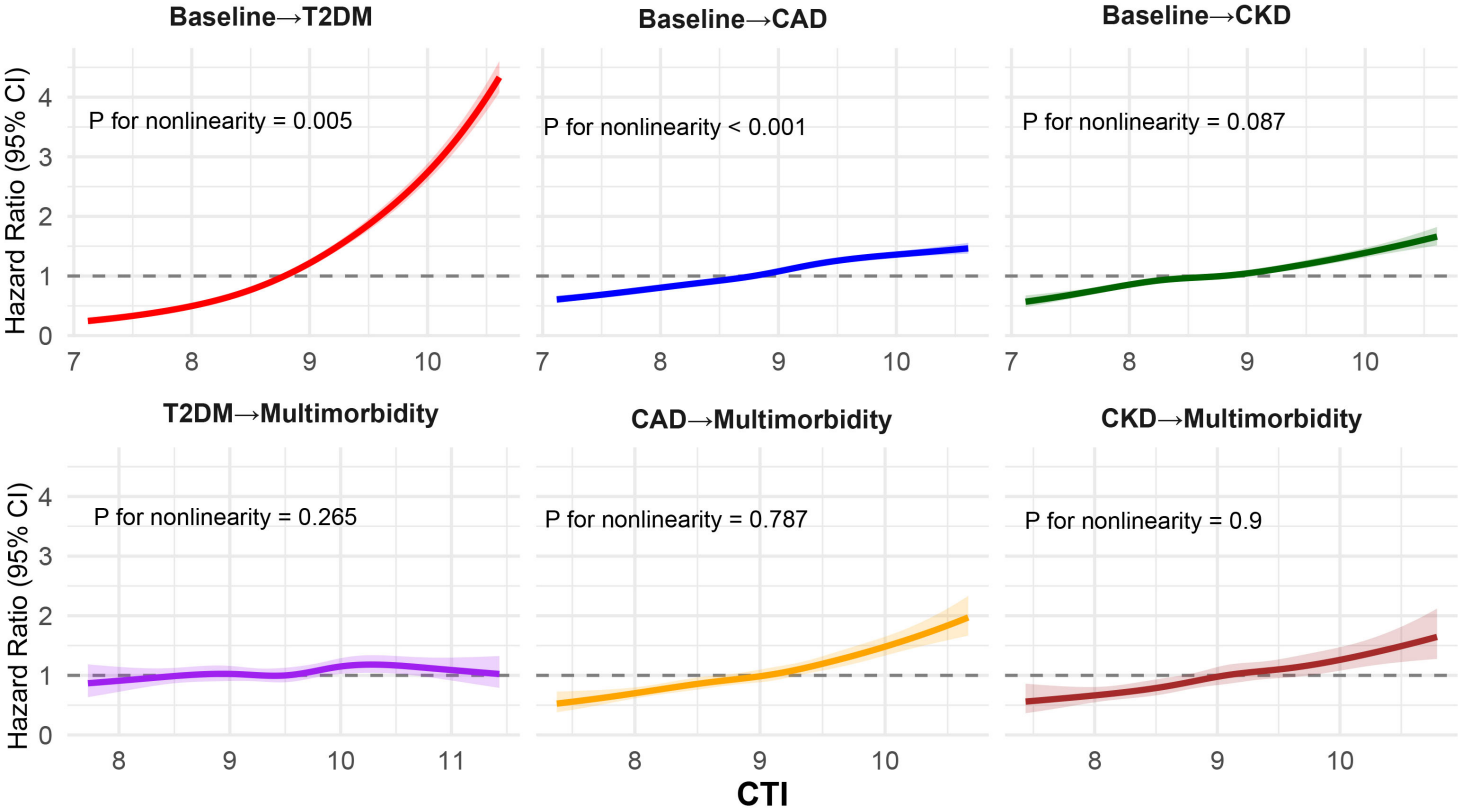
Receiver operating characteristic curves comparing the predictive performance of CRP, TyG, CTI, and the combination of CRP and TyG for incident T2DM, CAD, CKD, and multimorbidity. Area under the curve values with 95% confidence intervals are presented within each panel. Across all outcomes, CTI demonstrated superior discrimination compared with CRP, TyG, or their combination. CRP, C-reactive protein; TyG, triglyceride–glucose index; CTI, C-reactive protein–triglyceride–glucose index; T2DM, type 2 diabetes mellitus; CAD, coronary artery disease; CKD, chronic kidney disease.

ROC analyses assessed the predictive accuracy of TyG, CRP, CTI, and the combination of CRP and TyG for T2DM, CAD, CKD, and multimorbidity (**Figure 2**). For T2DM, CTI demonstrated the highest AUC at 0.753 (95% CI, 0.750–0.757), followed by the combination of CRP and TyG (0.736; 95% CI, 0.732–0.740), TyG (0.720; 95% CI, 0.716–0.724), and CRP (0.683; 95% CI, 0.679–0.687). Similar patterns were observed for CAD, with AUCs of 0.617 (95% CI, 0.613–0.620) for CTI, 0.609 (95% CI, 0.605–0.612) for the combination of CRP and TyG, 0.60 (95% CI, 0.597–0.604) for TyG, and 0.584 (95% CI, 0.581–0.588) for CRP.

For CKD, CTI again showed the highest AUC (0.623; 95% CI, 0.618–0.628), followed closely by CRP (0.618; 95% CI, 0.613–0.623), whereas the combination of CRP and TyG (0.608; 95% CI, 0.603–0.613) and TyG (0.585; 95% CI, 0.580–0.590) performed less well. For multimorbidity, CTI provided the greatest predictive value (0.716; 95% CI, 0.710–0.722), followed by the combination of CRP and TyG (0.698; 95% CI, 0.692–0.704), TyG (0.678; 95% CI, 0.671–0.684), and CRP (0.667; 95% CI, 0.660–0.673).

### Exposure–response across multistate transitions

**Figure 3:**
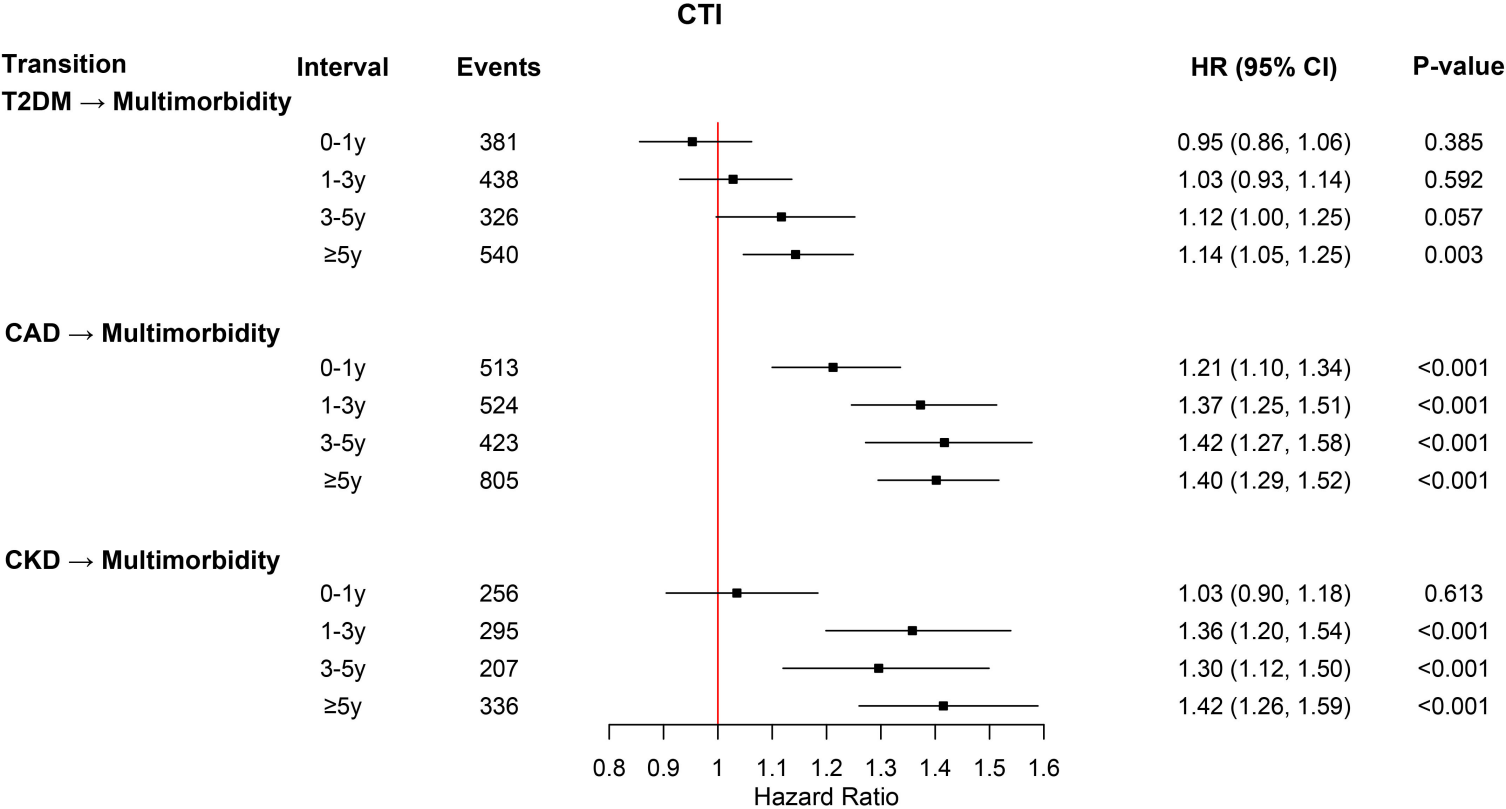
Restricted cubic spline analyses depicting associations of the CTI with transition-specific risks from baseline to CAD, T2DM, or CKD, and from each single disease to multimorbidity. Models were adjusted for age, sex, body mass index, race, physical activity, hypertension, cumulative dietary risk score, smoking status, alcohol consumption, lipid-lowering therapy, HbA1c, and fasting hours. Hazard ratios (solid lines) with 95% confidence intervals (shaded areas) are shown; P values reflect tests for nonlinearity.

RCS analyses within multistate models revealed heterogeneous exposure–response patterns of CTI across transitions (**Figure 3**). For baseline-to-disease transitions, most transitions showed significant departures from linearity (P < 0.05), indicating nonlinear associations between higher CTI levels and initial disease onset. In contrast, progression from single diseases to multimorbidity was generally compatible with linearity (all P > 0.05), suggesting proportional risk increases across increasing CTI levels.

### Temporal effects of CTI on transitions to multimorbidity

**Figure 4:**
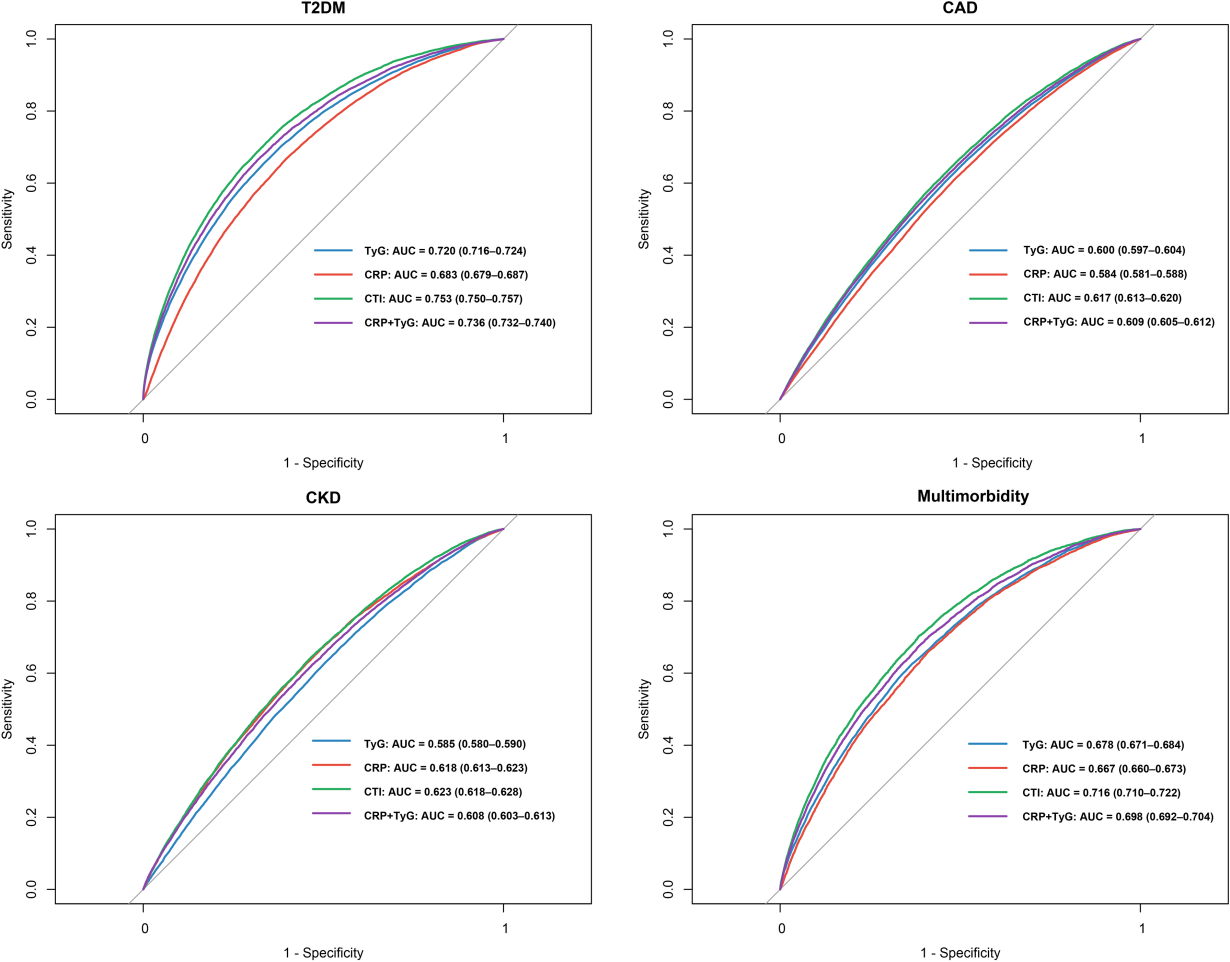
Hazard ratios (HRs) with 95% confidence intervals (CIs) for the association between the CTI index (per 1-SD increase) and risk of progression from T2DM, CAD, or CKD to multimorbidity across different follow-up intervals (0–1, 1–3, 3–5, and ≥5 years). Models were adjusted for age, sex, body mass index, race, physical activity, hypertension, cumulative dietary risk score, smoking status, alcohol consumption, lipid-lowering therapy, HbA1c, and fasting hours. HRs were estimated using piecewise Cox regression with time-split follow-up. CRP, C-reactive protein; TyG, triglyceride–glucose index; CTI, C-reactive protein–triglyceride–glucose index; T2DM, type 2 diabetes mellitus; CAD, coronary artery disease; CKD, chronic kidney disease.

CTI exhibited time-dependent associations with progression from single diseases to multimorbidity (**Figure 4**). For the transition from T2DM to multimorbidity, no associations were observed during 0-<1 or 1-<3 years, with borderline significance at 3–<5 years (HR, 1.12; 95% CI, 1.00-1.25; P = 0.057) and significant elevation thereafter (≥5 years: HR, 1.14; 95% CI, 1.05-1.25; P = 0.003). For the CAD-to-multimorbidity transition, risks increased progressively: 0-<1 year (HR, 1.21; 95% CI, 1.10-1.34), 1–<3 years (HR, 1.37; 95% CI, 1.25-1.51), 3-<5 years (HR, 1.42; 95% CI, 1.27-1.58), and ≥5 years (HR, 1.40; 95% CI, 1.29-1.52; all P<0.001). For the CKD-to-multimorbidity transition, no significant association emerged in the first year (P = 0.613), but risks were evident from 1-<3 years (HR, 1.36; 95% CI, 1.20-1.54) and persisted at 3-<5 years (HR, 1.30; 95% CI, 1.12-1.50) and ≥5 years (HR, 1.42; 95% CI, 1.26-1.59; all P<0.001).

### Subgroup and sensitivity analyses

In subgroup analyses, no significant interactions were observed for the T2DM-to-multimorbidity transition (all P for interaction >0.05; **Supplementary Figure 2**). For the CAD-to-multimorbidity transition, CTI showed effect modification by age, hypertension, and physical activity (all P for interaction <0.05; **Supplementary Figure 3**), with stronger associations in younger participants, those without hypertension, and individuals with lower physical activity. For the CKD-to-multimorbidity transition, significant interactions were observed for sex and hypertension (both P for interaction <0.05; **Supplementary Figure 4**), with stronger associations in women and in participants without hypertension. No other significant interactions were detected.

Six sensitivity analyses were conducted to assess the robustness of the results, and all were consistent with the main findings (**Supplementary Tables 4–9**).

## Discussion

In this large prospective cohort study, multistate models delineating trajectories within the CRD cluster yielded three principal findings. First, the CTI was robustly associated with elevated risks of incident CAD, T2DM, CKD, and multimorbidity, with predictive performance surpassing CRP, TyG, and their combination. Second, CTI exhibited trajectory-specific heterogeneity, with nonlinear dose–response relationships for transitions from baseline to single diseases, but predominantly linear associations for progression from single diseases to multimorbidity, and risks becoming more pronounced with cumulative time. Third, joint high exposure to CRP and TyG conferred the greatest relative risks, with significant additive interactions for T2DM onset and multimorbidity, underscoring the synergistic interplay between inflammatory and metabolic pathways in disease progression. Collectively, these findings establish CTI as a clinically relevant biomarker for refined risk stratification and targeted prevention.

CRP, TyG, and CTI were consistently associated with the onset of CAD, T2DM, and CKD, as well as with subsequent progression to multimorbidity. However, these associations were attenuated for TyG and CTI along the trajectory from T2DM to multimorbidity. This attenuation may reflect the preexisting burden of insulin resistance and metabolic dysregulation in patients with T2DM, which limits the incremental predictive value of TyG (9). Moreover, progression from T2DM to multimorbidity appears to be driven predominantly by vascular remodeling and renal hemodynamic disturbances rather than inflammatory pathways (6). Although CTI integrates metabolic and inflammatory signals, redundancy of metabolic risk and the secondary role of inflammation in this phase may attenuate its prognostic utility. These findings underscore mechanistic heterogeneity across disease stages and illustrate the value of multistate models in capturing dynamic cardiometabolic trajectories.

The CTI demonstrated superior predictive performance compared with CRP, TyG, or their combination, underscoring its ability to capture a broader spectrum of risk. This advantage likely arises from CTI’s integration of the bidirectional interplay between metabolic dysfunction and chronic inflammation. Insulin resistance promotes the release of proinflammatory cytokines such as TNF-α and IL-6, whereas inflammation impairs insulin signaling, creating a feed-forward cycle that amplifies cardiometabolic and renal injury (28). This immune–metabolic coupling is supported by mechanistic and translational evidence indicating that pro-inflammatory cytokines such as TNF-α and IL-6 can worsen insulin resistance through stress-kinase and NF-κB signaling, whereas insulin-resistant states can in turn promote low-grade inflammation, reinforcing a self-perpetuating cycle (29, 30). Joint exposure analyses reinforced this cumulative effect, showing graded increases in the risks of CAD, CKD, T2DM, and multimorbidity with concurrent elevations of CRP and TyG. By quantitatively capturing this synergistic pathophysiology, CTI offers greater clinical utility for risk stratification than individual biomarkers and may inform early identification and stage-specific preventive strategies.

Interaction analyses revealed synergistic effects of inflammation and metabolism on specific outcomes and trajectories. In conventional Cox regression, additive interactions indicated that concurrent elevations in CRP and TyG conferred risks for T2DM and multimorbidity that exceeded the sum of their individual effects. This observation is biologically plausible: insulin resistance promotes lipid dysregulation and oxidative stress, activating NF-κB and inducing CRP release, while inflammation reciprocally impairs insulin signaling and glucose uptake, together amplifying systemic metabolic stress and cardiorenal injury (31). Additional mechanisms, including inflammation-induced endothelial dysfunction, vascular stiffening, glomerular hyperfiltration, and fibrosis, likely further accelerate cross-organ progression in the presence of metabolic derangements (32). In multistate models, this inflammation–metabolism synergy was most evident in the transition from baseline to incident T2DM, underscoring its critical role in diabetes initiation. By contrast, interactions were attenuated during progression from single diseases to multimorbidity, suggesting that later stages are driven predominantly by vascular remodeling, renal hemodynamic disturbances, and established organ damage rather than inflammation–metabolism pathways. Conceptually, as disease advances, baseline risk is increasingly driven by fixed structural damage and the overall burden of disease management, resulting in risk “saturation” and reducing the apparent incremental contribution of circulating inflammatory–metabolic biomarkers, even if upstream biology remains active. Accordingly, we interpret the observed interaction as suggesting synergistic effects of inflammation and metabolic dysregulation along CRD trajectories, rather than providing definitive evidence for specific mechanistic pathways. Future studies that incorporate repeated biomarker assessments and clearer temporal sequencing, together with explicit causal frameworks and appropriate mediation–interaction approaches such as four-way decomposition, may help distinguish direct interaction from mediation-related (indirect) components of the interplay between CRP and TyG. Our study further revealed a cumulative escalation of CTI-associated risk during the progression from T2DM, CAD, or CKD to multimorbidity, with adverse effects amplifying over prolonged follow-up. These findings underscore the pivotal role of sustained metabolic–inflammatory injury in disease evolution. Mechanistically, persistent insulin resistance drives lipid dysregulation, impaired glucose metabolism, endothelial dysfunction, and arterial stiffening (33). Concurrently, chronic low-grade inflammation—through NF-κB activation and proinflammatory cytokine release—facilitates vascular remodeling and glomerular fibrosis, while oxidative stress and immune cell infiltration establish a cross-organ amplification loop between cardiovascular and renal systems, further destabilizing metabolic homeostasis (34, 35). These processes accumulate gradually, surpassing compensatory thresholds and accelerating the transition from single diseases to multimorbidity. Collectively, these results highlight CTI as a marker of long-term risk and reinforce the need for early interventions targeting metabolic–inflammatory pathways to disrupt disease trajectories.

Subgroup analyses revealed substantial heterogeneity in the associations between CTI and progression to multimorbidity, identifying potential susceptible populations. Among patients with CAD, higher risks were observed in younger individuals, those without hypertension, and those with low physical activity. Although younger and normotensive individuals are typically regarded as “relatively healthy,” the cumulative burden of metabolic–inflammatory injury may be more pronounced in these groups (36), suggesting that CTI captures risk signals more directly in the absence of conventional risk factors. One plausible interpretation is that, in these lower-burden subgroups, CTI better indexes upstream pathobiology related to inflammation–insulin resistance coupling, rather than downstream consequences of long-standing organ damage, yielding stronger relative associations. In individuals with low physical activity, the excess risk may reflect co-occurring adverse lifestyle patterns that worsen metabolic dysregulation and chronic inflammation, thereby amplifying CTI-associated risk. This is consistent with evidence that habitual physical activity improves insulin sensitivity and lowers systemic inflammation, including C-reactive protein, whereas physical inactivity promotes an inflammatory milieu and metabolic dysfunction that could magnify CTI-related risk (37). In CKD patients, women exhibited greater susceptibility than men, plausibly reflecting postmenopausal declines in estrogen that weaken anti-inflammatory protection and intensify the detrimental effects of metabolic–inflammatory pathways on renal and vascular systems (38). Experimental and clinical data support anti-inflammatory and vasculoprotective actions of estrogen signaling, and loss of estrogen after menopause has been linked to increased vascular inflammation and adverse cardiometabolic profiles, providing a plausible basis for the observed sex heterogeneity (39, 40). By contrast, in patients with T2DM, CTI effects were relatively consistent across subgroups, indicating that diabetes itself entails pervasive metabolic–inflammatory injury that diminishes incremental variation (28). Collectively, these findings suggest that clinical application of CTI should not only target conventionally high-risk groups but also account for these susceptible populations to achieve more precise risk stratification and individualized prevention.

This study has several strengths. First, the extended follow-up of more than 15 years enabled robust evaluation of long-term disease dynamics. Second, this is, to our knowledge, the first study to apply multistate modeling to comprehensively evaluate multimorbidity trajectories of CAD, CKD, and T2DM, thereby revealing heterogeneous effects across distinct transitions. Third, comparisons of CRP and TyG with CTI demonstrated CTI’s superior ability to capture integrated metabolic–inflammatory risk signals. Several limitations should also be acknowledged. Despite adjustment for multiple covariates, residual confounding from unmeasured genetic or environmental factors cannot be excluded. The cohort’s predominantly European ancestry may limit the generalizability of findings to other populations. Moreover, CTI was derived from baseline laboratory values only, precluding assessment of temporal changes that may further influence disease trajectories.

## Conclusions

CRP, TyG, and CTI were strongly associated with incident CRD cluster, with CTI showing superior predictive accuracy over CRP, TyG, or their combination. CTI demonstrated trajectory specific heterogeneity, with nonlinear associations for transitions from disease-free states to single conditions but largely linear patterns for progression to multimorbidity, and risks amplifying cumulatively over time. Concurrent elevations in CRP and TyG conferred the greatest risks, with significant additive interactions for T2DM onset and multimorbidity, underscoring the synergistic interplay between metabolic dysfunction and inflammation in CRD cluster progression.

## Data Availability

The original contributions presented in the study are included in the article/**Supplementary Material**. Further inquiries can be directed to the corresponding author.
