# Supplementary material for: Associations of C-reactive protein, triglyceride–glucose index, and the C-reactive protein–triglyceride glucose index with multistate trajectories in the cardiovascular–renal–diabetes cluster
Source: Front Endocrinol (Lausanne). 2026 Mar 16;17:1758467. doi: 10.3389/fendo.2026.1758467 (PMC13033564; doi:10.3389/fendo.2026.1758467)

Supplement

C-reactive Protein–Triglyceride–Glucose Index: A Novel Inflammatory–Metabolic Predictor of Multistate Progression in the Cardiovascular–Renal–Diabetes Cluster

Hui Li, Liuyu Chen, Mengyi Wang, Wenke Cheng, Zhongyan Du, Yuli Huang

Table S1. Diagnostic codes for diseases

Diseases	ICD-9 diagnosis	ICD-10 diagnosis	Self-report [a]	Primary Care [b]	Medication [c] / Operation code, self-reported [d]
Hypertension	401, 4010, 4011, 4019, 402, 4020, 4021, 4029, 403, 4030, 4031, 4039, 404, 4040, 4041, 4049, 405, 4050, 4051, 4059	I10-I13, I15	20002	Category 3000	Blood pressure medication / NA
Diabetes	2500, 25000, 25001, 25009, 2501, 25011, 25019, 2503, 2504, 2505, 25099	E10-E14	20002	Category 3000	Insulin/ NA
CAD	410, 4109, 411, 4119, 412, 4129, 413, 4139, 4140, 4148, 4149	I20-I25	20002	Category 3000	NA / Coronary angioplasty (PTCA) +/- stent; Coronary artery bypass grafts (CABG); Triple heart bypass
CKD	585.x, 585.9	N18	20002	Category 3000	eGFR below 60 mL/min/1.73m^2 or albuminuria above 3 mg/mmol at baseline.
Stroke	430-434, 436	I60-64,Field 40001, Field 40002	20002	Category 3000	NA
Heart failure	428, 4280, 4281, 4289	I50	20002	Category 3000	NA
cardiomyopathy	422, 4229, 425, 4251, 4254	I40-I43	20002	Category 3000	NA
Heart valve disease	394, 3940, 3942, 3949, 395, 3951, 3959, 396, 3969, 424, 4240, 4241, 4243, 4249	I05-I09, I34-I38	20002	Category 3000	NA / Aortic valve repair/replacement; Mitral valve repair/replacement; Other valve repair/replacement
Arrhythmia	426, 4260, 4261, 4263, 4264, 4265, 4266, 4267, 4269, 427, 4270, 4271, 4273, 4274, 4276, 4278, 4279	I44, I45, I47-I49	20002	Category 3000	NA / Pacemaker; Defibrillator insertion; Cardiac ablation

ICD: International Classification of Disease. NA, not applicable.

[a] ICD-10 codes in the Hospital inpatient data and Death Register records

[b] 20002 is the data code used in UK Biobank: Non-cancer illness code, self-reported.

[c] This category contains data on primary care data recorded by health professionals working at general practices.

[d] Data-field 6153 and 6177.

[e] Data-Field 20004

Table S2. The calculation method for cumulative dietary risk

Variable	Categories reported from the touch-screen questionnaire	Binary Variables
Fruit & vegetables (regrouped from fruit, dried fruit & Vegetable)	Serving/day	≥5 serving/day (Ref.)<br><5 serving/day
Total fish intake (regrouped from Both total non-oily fish and oily fish)	Never<br>Less than once a week<br>Once a week<br>2-4 times a week<br>5-6 times a week<br>Once or more daily	≥2 times a week (at least once a week of each category)<br>(Ref.)<br>< once a week of each one
Processed meat intake	Never<br>Less than once a week<br>Once a week<br>2-4 times a week<br>5-6 times a week<br>Once or more daily	≤Once a week<br>(Ref.)<br>> Once a week
Red meat (regrouped from beef, pork and lamb)	Never<br>Less than once a week<br>Once a week<br>2-4 times a week<br>5-6 times a week<br>Once or more daily	≤Once a week (Ref.)<br>>Once a week
Milk type used	Full cream<br>Semi-skimmed<br>Skimmed<br>Soya<br>another type of milk<br>Never rarely have milk	Semi-skimmed/skimmed (Ref.)<br>Full cream/ another type of milk/ never rarely have milk
Spread type	Never/rarely<br>Butter<br>Other type/ margarine<br>Flora pro-active/benecol	Never/rarely (Ref.)<br>Another selection
Cereal intake	Bowls/week	>5 bowls (Ref)<br>≤5 bowls
Salt added to food	Never/rarely<br>Sometimes<br>Usually<br>Always	Never/rarely (Ref.)<br>Another selection
Water intake	Glasses/day	≥6 glasses (Ref.)<br><6 glasses

Table S3. Covariate selection based on change-in-estimate and statistical significance criteria.

Variable	Beta change percent (%)	P value	Include		Beta change percent (%)	P value	Include
**CTI and CAD**				**CTI and CKD**			
Age	12.92	< 0.001	Yes	Age	16.79	< 0.001	Yes
Sex	10.35	< 0.001	Yes	Sex	1.57	0.998	No
BMI	8.63	< 0.001	Yes	BMI	18.26	< 0.001	Yes
Diet score	1.99	< 0.001	Yes	Diet score	0.24	0.192	No
Hypertension	12.08	< 0.001	Yes	Hypertension	18.44	< 0.001	Yes
Physical activity	0.12	< 0.001	Yes	Physical activity	2.15	< 0.001	Yes
Lipid-lowering agents	4.72	< 0.001	Yes	Lipid-lowering agents	6.06	< 0.001	Yes
Race	0.08	< 0.001	Yes	Race	0.07	0.002	Yes
HbA1c	7.47	< 0.001	Yes	HbA1c	8.87	< 0.001	Yes
Fasting	0.33	< 0.001	Yes	Fasting	0.36	< 0.001	Yes
Alcohol consumption	0.59	< 0.001	Yes	Alcohol	1.19	< 0.001	Yes
Smoking status	3.35	< 0.001	Yes	Smoking	0.2	< 0.001	Yes
**CTI and T2DM**				**CTI and multimorbidity**			
Age	0.64	< 0.001	Yes	Age	3.57	< 0.001	Yes
Sex	1.05	< 0.001	Yes	Sex	2.63	< 0.001	Yes
BMI	18.58	< 0.001	Yes	BMI	16.32	< 0.001	Yes
Diet score	0.97	< 0.001	Yes	Diet score	0.99	< 0.001	Yes
Hypertension	4.58	< 0.001	Yes	Hypertension	9.06	< 0.001	Yes
Physical activity	1.14	< 0.001	Yes	Physical activity	1.59	< 0.001	Yes
Lipid-lowering agents	1.47	< 0.001	Yes	Lipid-lowering agents	3.07	< 0.001	Yes
Race	0.22	< 0.001	Yes	Race	0.07	< 0.001	Yes
HbA1c	12.57	< 0.001	Yes	HbA1c	12.23	< 0.001	Yes
Fasting	0.48	< 0.001	Yes	Fasting	0.46	< 0.001	Yes
Alcohol consumption	0.75	< 0.001	Yes	Alcohol consumption	0.99	< 0.001	Yes
Smoking status	0.83	< 0.001	Yes	Smoking status	1.75	< 0.001	Yes

CTI, C-reactive protein-triglyceride-glucose index. CAD, coronary artery disease. T2DM, type 2 diabetes mellitus. CKD, chronic kidney disease. BMI, body mass index.

Beta change percent (%) was calculated as the absolute percent change in the Cox regression coefficient (β, i.e., log hazard ratio) for CTI when each covariate was added individually to the unadjusted CTI-only Cox model: $100 \times |(\beta_{(CTI+covariate)} - \beta_{(CTI\text{-}only)}) / \beta_{(CTI\text{-}only)}|$.

P values were obtained from univariable Cox models for each covariate.

Table S4. Associations of CRP, TyG, and CTI with dynamic transitions within the cardiovascular–renal–diabetes cluster, evaluated using conventional Cox regression and multistate models after excluding participants who developed CAD, T2DM, or CKD within the first two years of follow-up.

	Case	Proportion (%)	HR (95% CI)	*P*-value	HR (95% CI)	*P*-value	HR (95% CI)	*P*-value
**Traditional Cox model**								
Per 1-SD increase			**CRP**		**TyG**		**CTI**	
T2DM	14185	4.3%	1.09 (1.08, 1.11)	<0.001	1.63 (1.60, 1.65)	<0.001	1.81 (1.78, 1.84)	<0.001
CAD	22003	6.7%	1.07 (1.06, 1.08)	<0.001	1.14 (1.12, 1.16)	<0.001	1.23 (1.21, 1.24)	<0.001
CKD	10426	3.2%	1.10 (1.08, 1.11)	<0.001	1.09 (1.06, 1.11)	<0.001	1.23 (1.20, 1.25)	<0.001
Multimorbidity	5470	1.7%	1.12 (1.11, 1.4)	<0.001	1.37 (1.33, 1.41)	<0.001	1.57 (1.52, 1.62)	<0.001
**Multi-state model**								
Per 1-SD increase			**CRP**		**TyG**		**CTI**	
Baseline → T2DM	12021	3.7%	1.11 (1.10, 1.12)	<0.001	1.80 (1.77, 1.84)	<0.001	2.04 (2.0, 2.09)	<0.001
Baseline → CAD	19322	5.9%	1.08 (1.06, 1.09)	<0.001	1.14 (1.13, 1.16)	<0.001	1.24 (1.22, 1.26)	<0.001
Baseline → CKD	8069	2.5%	1.10 (1.09, 1.12)	<0.001	1.06 (1.06, 1.11)	<0.001	1.20 (1.17, 1.23)	<0.001
T2DM → Multimorbidity	1453	12.2%	1.08 (1.04,1.11)	<0.001	1.0 (0.94, 1.04)	0.732	1.06 (1.0, 1.12)	0.053
CAD → Multimorbidity	1841	10.4%	1.09 (1.05, 1.12)	<0.001	1.16 (1.10, 1.22)	<0.001	1.30 (1.23, 1.38)	<0.001
CKD → Multimorbidity	914	11.5%	1.11 (1.06, 1.15)	<0.001	1.18 (1.10, 1.27)	<0.001	1.31 (1.21, 1.42)	<0.001

CRP, C-reactive protein; TyG, triglyceride–glucose index; CTI, C-reactive protein–triglyceride–glucose index; T2DM, type 2 diabetes mellitus; CAD, coronary artery disease; CKD, chronic kidney disease.

Traditional Cox models estimated the overall associations of a 1-SD increase (Z-standardized) in CRP, TyG, and CTI with the incidence of T2DM, CAD, CKD, and multimorbidity.

Multi-state models were then used to evaluate transition-specific hazards from baseline to each disease state

All models were adjusted for age, sex, body mass index , race, physical activity, hypertension, cumulative dietary-risk score, smoking status, alcohol consumption, lipid-lowering drugs, HbA1c, and fasting hours.

Table S5. Associations of CRP, TyG, and CTI with dynamic transitions in the cardiovascular–renal–diabetes cluster, based on multiple imputation (5 datasets) with Rubin's rules.

	Case	Proportion (%)	HR (95% CI)	*P*-value	HR (95% CI)	*P*-value	HR (95% CI)	*P*-value
**Traditional Cox model**								
Per 1-SD increase			**CRP**		**TyG**		**CTI**	
T2DM	15608	4.7%	1.10 (1.09, 1.11)	<0.001	1.67 (1.65, 1.70)	<0.001	1.87 (1.84, 1.90)	<0.001
CAD	24676	7.4%	1.07 (1.06, 1.08)	<0.001	1.14 (1.13, 1.16)	<0.001	1.23 (1.21, 1.25)	<0.001
CKD	11763	3.5%	1.10 (1.09, 1.11)	<0.001	1.10 (1.08, 1.12)	<0.001	1.24 (1.21, 1.27)	<0.001
Multimorbidity	6350	1.9%	1.13 (1.11, 1.14)	<0.001	1.40 (1.36, 1.43)	<0.001	1.61 (1.56, 1.65)	<0.001
**Multi-state model**								
Per 1-SD increase			**CRP**		**TyG**		**CTI**	
Baseline → T2DM	13063	3.93%	1.10 (1.09, 1.11)	<0.001	1.69 (1.66, 1.72)	<0.001	1.90 (1.86, 1.93)	<0.001
Baseline → CAD	21677	6.52%	1.07 (1.06, 1.08)	<0.001	1.14 (1.12, 1.16)	<0.001	1.23 (1.21, 1.25)	<0.001
Baseline → CKD	9077	2.73%	1.10 (1.09, 1.11)	<0.001	1.08 (1.06, 1.10)	<0.001	1.22 (1.19, 1.25)	<0.001
T2DM → Multimorbidity	1685	13%	1.06 (1.03,1.09)	<0.001	1.0 (0.95, 1.05)	0.904	1.06 (1.0, 1.12)	0.055
CAD → Multimorbidity	2265	11.32%	1.08 (1.05, 1.11)	<0.001	1.22 (1.17, 1.28)	<0.001	1.35 (1.29, 1.42)	<0.001
CKD → Multimorbidity	1094	12.17%	1.08 (1.04, 1.12)	<0.001	1.17 (1.09, 1.25)	<0.001	1.28 (1.19, 1.37)	<0.001

CRP, C-reactive protein; TyG, triglyceride–glucose index; CTI, C-reactive protein–triglyceride–glucose index; T2DM, type 2 diabetes mellitus; CAD, coronary artery disease; CKD, chronic kidney disease.

Traditional Cox models estimated the overall associations of a 1-SD increase (Z-standardized) in CRP, TyG, and CTI with the incidence of T2DM, CAD, CKD, and multimorbidity.

Multi-state models were then used to evaluate transition-specific hazards from baseline to each disease state

All models were adjusted for age, sex, body mass index , race, physical activity, hypertension, cumulative dietary-risk score, smoking status, alcohol consumption, lipid-lowering drugs, HbA1c, and fasting hours.

Table S6. Associations of CRP, TyG, and CTI with dynamic transitions in the cardiovascular–renal–diabetes cluster.

	**HR (95% CI)**	***P*-value**	**HR (95% CI)**	***P*-value**	**HR (95% CI)**	***P*-value**
**Traditional Cox model**						
Per 1-SD increase	**CRP**		**TyG**		**CTI**	
T2DM	1.19 (1.17, 1.20)	<0.001	1.63 (1.60, 1.66)	<0.001	1.72 (1.68, 1.75)	<0.001
CAD	1.10 (1.09, 1.12)	<0.001	1.13 (1.12, 1.15)	<0.001	1.21 (1.19, 1.23)	<0.001
CKD	1.13 (1.11, 1.15)	<0.001	1.08 (1.06, 1.10)	<0.001	1.19 (1.17, 1.22)	<0.001
Multimorbidity	1.20 (1.17, 1.22)	<0.001	1.35 (1.31, 1.39)	<0.001	1.51 (1.46, 1.56)	<0.001
**Multi-state model**						
Per 1-SD increase	**CRP**		**TyG**		**CTI**	
Baseline → T2DM	1.10 (1.08, 1.11)	<0.001	1.69 (1.66, 1.72)	<0.001	1.88 (1.85, 1.92)	<0.001
Baseline → CAD	1.07 (1.06, 1.08)	<0.001	1.14 (1.12, 1.16)	<0.001	1.23 (1.21, 1.25)	<0.001
Baseline → CKD	1.10 (1.08, 1.11)	<0.001	1.08 (1.06, 1.11)	<0.001	1.22 (1.19, 1.25)	<0.001
T2DM → Multimorbidity	1.08 (1.03, 1.12)	<0.001	1.0 (0.94, 1.05)	0.902	1.05 (1.0, 1.12)	0.057
CAD → Multimorbidity	1.09 (1.06, 1.13)	<0.001	1.21 (1.16, 1.26)	<0.001	1.32 (1.26, 1.38)	<0.001
CKD → Multimorbidity	1.10 (1.05, 1.15)	<0.001	1.15 (1.09, 1.23)	<0.001	1.26 (1.18, 1.34)	<0.001

The analytic dataset was restricted to the 2.5th–97.5th percentiles of the exposure distribution to minimize the influence of outliers and to evaluate the robustness of associations between CTI and disease transitions.

CRP, C-reactive protein; TyG, triglyceride–glucose index; CTI, C-reactive protein–triglyceride–glucose index; T2DM, type 2 diabetes mellitus; CAD, coronary artery disease; CKD, chronic kidney disease.

Traditional Cox models estimated the overall associations of a 1-SD increase (Z-standardized) in CRP, TyG, and CTI with the incidence of T2DM, CAD, CKD, and multimorbidity.

Multi-state models were then used to evaluate transition-specific hazards from baseline to each disease state

All models were adjusted for age, sex, body mass index , race, physical activity, hypertension, cumulative dietary-risk score, smoking status, alcohol consumption, lipid-lowering drugs, HbA1c, and fasting hours.

Table S7. Complete-Case Analyses of CRP, TyG, and CTI with Multistate Trajectories in the Cardiovascular–Renal–Diabetes Cluster.

	Case	Proportion (%)	HR (95% CI)	*P*-value	HR (95% CI)	*P*-value	HR (95% CI)	*P*-value
**Traditional Cox model**								
Per 1-SD increase			**CRP**		**TyG**		**CTI**	
T2DM	13612	4.1%	1.09 (1.08, 1.11)	<0.001	1.68 (1.65, 1.70)	<0.001	1.87 (1.83, 1.90)	<0.001
CAD	22066	6.6%	1.07 (1.06, 1.08)	<0.001	1.15 (1.13, 1.16)	<0.001	1.23 (1.21, 1.25)	<0.001
CKD	10482	3.1%	1.10 (1.09, 1.11)	<0.001	1.09 (1.07, 1.12)	<0.001	1.23 (1.20, 1.26)	<0.001
Multimorbidity	5559	1.7%	1.12 (1.10, 1.4)	<0.001	1.39 (1.36, 1.43)	<0.001	1.59 (1.54, 1.64)	<0.001
**Multi-state model**								
Per 1-SD increase			**CRP**		**TyG**		**CTI**	
Baseline → T2DM	11409	6.45%	1.10 (1.08, 1.11)	<0.001	1.70 (1.67, 1.73)	<0.001	1.89 (1.85, 1.93)	<0.001
Baseline → CAD	19441	2.69%	1.06 (1.05, 1.08)	<0.001	1.14 (1.12, 1.16)	<0.001	1.23 (1.20, 1.25)	<0.001
Baseline → CKD	8119	3.79%	1.10 (1.09, 1.12)	<0.001	1.08 (1.05, 1.10)	<0.001	1.22 (1.19, 1.25)	<0.001
T2DM → Multimorbidity	1461	11.13%	1.06 (1.02,1.09)	<0.001	0.99 (0.94, 1.05)	0.823	1.06 (0.99, 1.12)	0.085
CAD → Multimorbidity	2002	11.79%	1.08 (1.04, 1.11)	<0.001	1.21 (1.15, 1.27)	<0.001	1.33 (1.26, 1.40)	<0.001
CKD → Multimorbidity	948	12.91%	1.08 (1.05, 1.12)	<0.001	1.17 (1.09, 1.26)	<0.001	1.28 (1.19, 1.38)	<0.001

CRP, C-reactive protein; TyG, triglyceride–glucose index; CTI, C-reactive protein–triglyceride–glucose index; T2DM, type 2 diabetes mellitus; CAD, coronary artery disease; CKD, chronic kidney disease.

Traditional Cox models estimated the overall associations of a 1-SD increase (Z-standardized) in CRP, TyG, and CTI with the incidence of T2DM, CAD, CKD, and multimorbidity.

Multi-state models were then used to evaluate transition-specific hazards from baseline to each disease state

All models were adjusted for age, sex, body mass index , race, physical activity, hypertension, cumulative dietary-risk score, smoking status, alcohol consumption, lipid-lowering drugs, HbA1c, and fasting hours.

Table S8. Associations of CRP, TyG, and CTI with dynamic transitions within the cardiovascular–renal–diabetes cluster with additional adjustment for low-density lipoprotein cholesterol and antihypertensive therapy.

	Case	Proportion (%)	HR (95% CI)	*P*-value	HR (95% CI)	*P*-value	HR (95% CI)	*P*-value
**Traditional Cox model**								
Per 1-SD increase			**CRP**		**TyG**		**CTI**	
T2DM	15608	4.7%	1.10 (1.09, 1.11)	<0.001	1.69 (1.66, 1.72)	<0.001	1.88 (1.85, 1.91)	<0.001
CAD	24676	7.4%	1.08 (1.07, 1.09)	<0.001	1.08 (1.07, 1.10)	<0.001	1.18 (1.16, 1.20)	<0.001
CKD	11763	3.5%	1.10 (1.08, 1.11)	<0.001	1.11 (1.09, 1.14)	<0.001	1.25 (1.23, 1.28)	<0.001
Multimorbidity	6350	1.9%	1.13 (1.11, 1.4)	<0.001	1.38 (1.34, 1.42)	<0.001	1.58 (1.54, 1.63)	<0.001
**Multi-state model**								
Per 1-SD increase			**CRP**		**TyG**		**CTI**	
Baseline → T2DM	13063	3.93%	1.10 (1.09, 1.11)	<0.001	1.72 (1.69, 1.75)	<0.001	1.92 (1.88, 1.95)	<0.001
Baseline → CAD	21677	6.52%	1.08 (1.06, 1.09)	<0.001	1.07 (1.06, 1.09)	<0.001	1.17 (1.15, 1.19)	<0.001
Baseline → CKD	9077	2.73%	1.10 (1.08, 1.11)	<0.001	1.01 (1.07, 1.13)	<0.001	1.24 (1.21, 1.27)	<0.001
T2DM → Multimorbidity	1685	13%	1.06 (1.02,1.09)	<0.001	0.99 (0.94, 1.04)	0.597	1.05 (0.99, 1.11)	0.119
CAD → Multimorbidity	2265	11.32%	1.08 (1.05, 1.11)	<0.001	1.22 (1.17, 1.28)	<0.001	1.36 (1.29, 1.43)	<0.001
CKD → Multimorbidity	1094	12.17%	1.08 (1.05, 1.12)	<0.001	1.16 (1.09, 1.25)	<0.001	1.27 (1.19, 1.37)	<0.001

CRP, C-reactive protein; TyG, triglyceride–glucose index; CTI, C-reactive protein–triglyceride–glucose index; T2DM, type 2 diabetes mellitus; CAD, coronary artery disease; CKD, chronic kidney disease.

Traditional Cox models estimated the overall associations of a 1-SD increase (z-standardized) in CRP, TyG, and CTI with the incidence of T2DM, CAD, CKD, and multimorbidity.

Multi-state models were then used to evaluate transition-specific hazards from baseline to each disease state

All models were adjusted for age, sex, body mass index , race, physical activity, hypertension, cumulative dietary-risk score, smoking status, alcohol consumption, lipid-lowering drugs, HbA1c, fasting hours, low-density lipoprotein cholesterol and antihypertensive therapy.

Table S9. Associations of CRP, TyG, and CTI with dynamic transitions within the cardiovascular–renal–diabetes cluster including a direct baseline-to-multimorbidity pathway.

	HR (95% CI)	*P*-value	HR (95% CI)	*P*-value	HR (95% CI)	*P*-value
**Multi-state model**						
Per 1-SD increase	**CRP**		**TyG**		**CTI**	
Baseline → T2DM	1.10 (1.08, 1.11)	<0.001	1.69 (1.66, 1.72)	<0.001	1.88 (1.84, 1.91)	<0.001
Baseline → CAD	1.07 (1.05, 1.08)	<0.001	1.14 (1.12, 1.16)	<0.001	1.23 (1.20, 1.24)	<0.001
Baseline → CKD	1.10 (1.08, 1.11)	<0.001	1.08 (1.05, 1.11)	<0.001	1.22 (1.19, 1.25)	<0.001
Baseline → Multimorbidity	1.10 (1.06, 1.09)	<0.001	1.49 (1.41,1.57)	<0.001	1.63 (1.53, 1.73)	<0.001
T2DM → Multimorbidity	1.06 (1.02, 1.09)	<0.001	1.0 (0.95, 1.05)	0.902	1.06 (1.0, 1.12)	0.057
CAD → Multimorbidity	1.08 (1.05, 1.11)	<0.001	1.22 (1.16, 1.27)	<0.001	1.35 (1.28, 1.42)	<0.001
CKD → Multimorbidity	1.08 (1.04, 1.12)	<0.001	1.17 (1.09, 1.24)	<0.001	1.28 (1.19, 1.37)	<0.001

CRP, C-reactive protein; TyG, triglyceride–glucose index; CTI, C-reactive protein–triglyceride–glucose index; T2DM, type 2 diabetes mellitus; CAD, coronary artery disease; CKD, chronic kidney disease.

Traditional Cox models estimated the overall associations of a 1-SD increase (Z-standardized) in CRP, TyG, and CTI with the incidence of T2DM, CAD, CKD, and multimorbidity.

Multi-state models were then used to evaluate transition-specific hazards from baseline to each disease state

All models were adjusted for age, sex, body mass index , race, physical activity, hypertension, cumulative dietary-risk score, smoking status, alcohol consumption, lipid-lowering drugs, HbA1c, and fasting hours.

Figure S1. Directed acyclic graph of the link between CRP, TyG index, CTI and the incidence of CAD/T2DM/CKD.

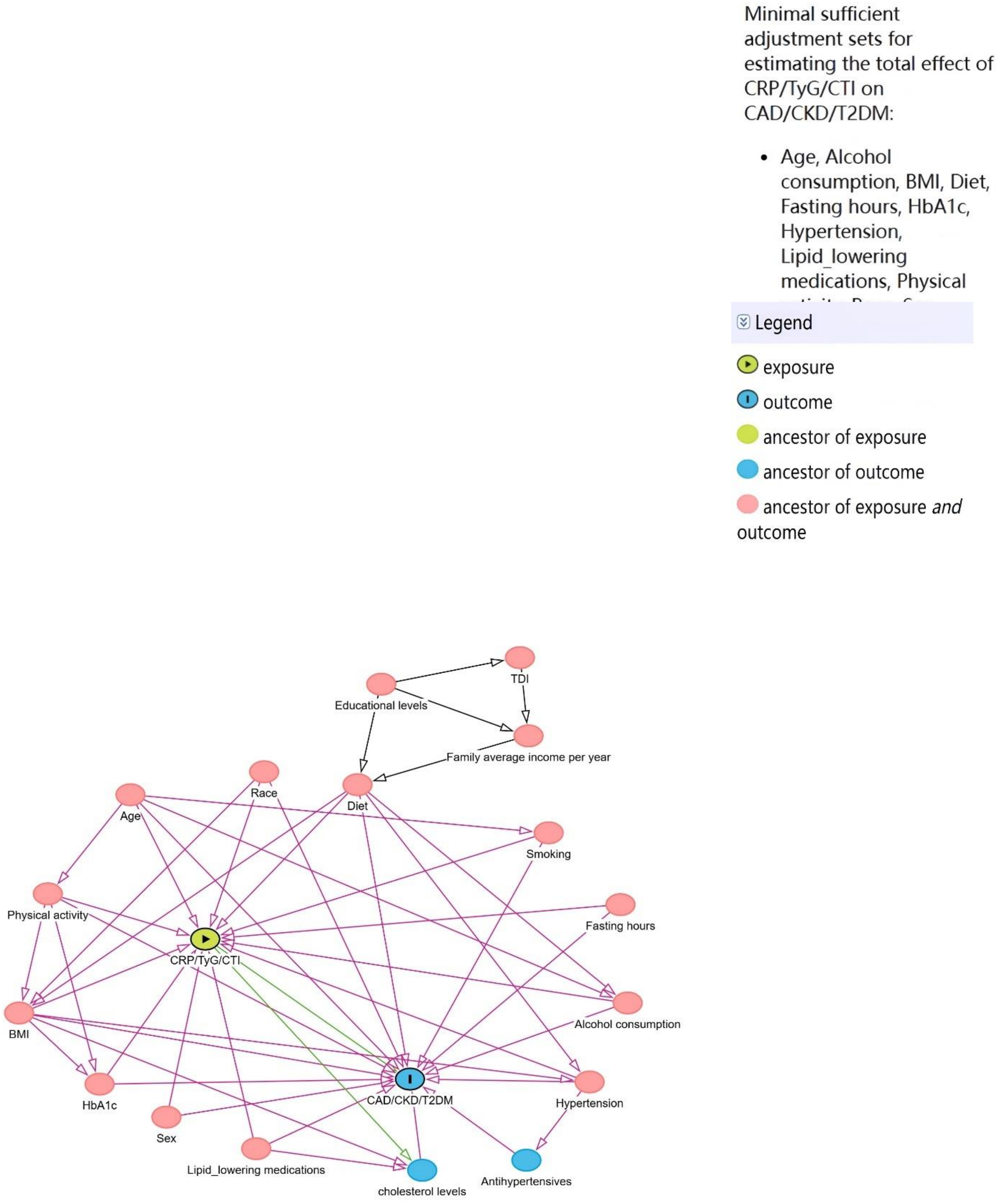

Abbreviations: CRP, C-reactive protein; CTI, C-reactive protein-triglyceride-glucose index; TyG,Triglyceride-glucose index. T2DM, type 2 diabetes mellitus; CAD, coronary artery disease; CKD, chronic kidney disease;

In the DAG diagram, green ovals represent the exposure, and blue ovals represent the outcome. Light green and light blue nodes indicate variables that are ancestors of the exposure and outcome, respectively. Pink nodes are shared ancestors of both exposure and outcome. Causal paths are shown as green arrows, and potential biasing paths are marked in purple.

Notes:
The graph was created with the help of DAGitty.net (www.dagitty.net). Minimally sufficient adjustment set: age, sex, BMI, race, physical activity, hypertension, cumulative dietary risk score, smoking status, alcohol consumption, lipid-lowering therapy, HbA1c, and fasting hours.

Figure S2. Associations between CTI and the transition from T2DM to multimorbidity, stratified by potential effect modifiers.
Models were adjusted for age, sex, body mass index , race, physical activity, hypertension, cumulative dietary-risk score, smoking status, alcohol consumption, lipid-lowering drugs, HbA1c, and fasting hours.
Hazard ratios (HRs) with 95% confidence intervals (CIs) represent the risk associated with a 1-SD increase in CTI. Interaction terms were tested by including cross-product terms (CTI × stratification variable) in the fully

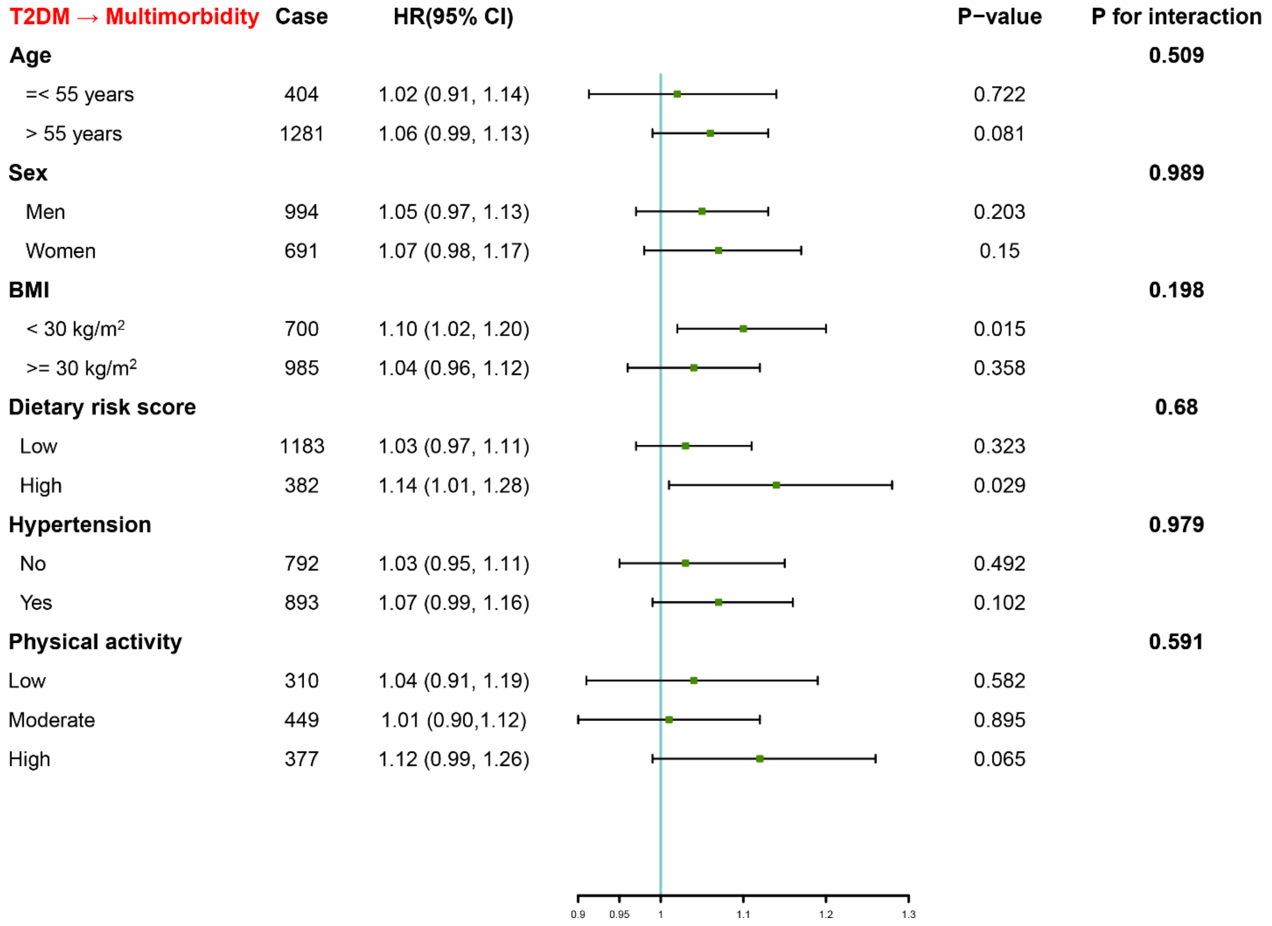

adjusted models. T2DM, type 2 diabetes mellitus.

Figure S3. Associations between CTI and the transition from CAD to multimorbidity, stratified by potential effect modifiers.

Models were adjusted for age, sex, body mass index , race, physical activity, hypertension, cumulative dietary-risk score, smoking status, alcohol consumption, lipid-lowering drugs, HbA1c, and fasting hours.

Hazard ratios (HRs) with 95% confidence intervals (CIs) represent the risk associated with a 1-SD increase in CTI. Interaction terms were tested by including cross-product terms (CTI × stratification variable) in the fully adjusted models. CAD, coronary artery disease;

CAD → Multimorbidity	Case	HR(95% CI)	P−value	P for interaction
**Age**				**0.002**
=< 55 years	433	1.48 (1.32, 1.65)	<0.001	
> 55 years	1832	1.24 (1.18, 1.32)	<0.001	
**Sex**				**0.325**
Men	1425	1.35 (1.26, 1.43)	<0.001	
Women	840	1.28 (1.18, 1.40)	<0.001	
**BMI**				**0.539**
< 30 kg/m^2	1425	1.38 (1.30, 1.47)	<0.001	
>= 30 kg/m^2	840	1.25 (1.15, 1.37)	<0.001	
**Dietary risk score**				**0.199**
Low	1637	1.26 (1.18, 1.33)	<0.001	
High	499	1.33 (1.20, 1.48)	<0.001	
**Hypertension**				**0.023**
No	1215	1.39 (1.29, 1.48)	<0.001	
Yes	1050	1.22 (1.14, 1.32)	<0.001	
**Physical activity**				**0.003**
Low	344	1.48 (1.30, 1.69)	<0.001	
Moderate	637	1.36 (1.24, 1.50)	<0.001	
High	644	1.23 (1.12, 1.35)	<0.001	

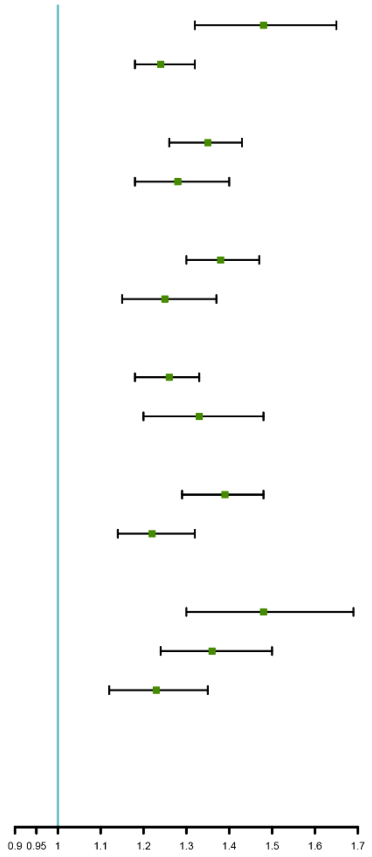

Figure S4. Associations between CTI and the transition from CKD to multimorbidity, stratified by potential effect modifiers.

Models were adjusted for age, sex, body mass index , race, physical activity, hypertension, cumulative dietary-risk score, smoking status, alcohol consumption, lipid-lowering drugs, HbA1c, and fasting hours.

Hazard ratios (HRs) with 95% confidence intervals (CIs) represent the risk associated with a 1-SD increase in CTI. Interaction terms were tested by including cross-product terms (CTI × stratification variable) in the fully adjusted models. CKD, chronic kidney disease.

CKD → Multimorbidity	Case	HR(95% CI)	P−value	P for interaction
**Age**				**0.06**
=< 55 years	170	1.44 (1.21,1.71)	<0.001	
> 55 years	924	1.26 (1.16,1.36)	<0.001	
**Sex**				**0.018**
Men	577	1.19 (1.08, 1.31)	<0.001	
Women	517	1.36 (1.22, 1.51)	<0.001	
**BMI**				**0.543**
< 30 kg/m^2	667	1.32 (1.21, 1.43)	<0.001	
>= 30 kg/m^2	427	1.27 (1.12, 1.44)	<0.001	
**Dietary risk score**				**0.492**
Low	810	1.27 (1.17, 1.38)	<0.001	
High	214	1.35 (1.14, 1.59)	<0.001	
**Hypertension**				**<0.001**
No	488	1.43 (1.28, 1.59)	<0.001	
Yes	606	1.16 (1.05, 1.28)	0.002	
**Physical activity**				**0.63**
Low	161	1.35 (1.13, 1.61)	<0.001	
Moderate	293	1.29 (1.12, 1.49)	<0.001	
High	283	1.21 (1.05, 1.39)	0.007	

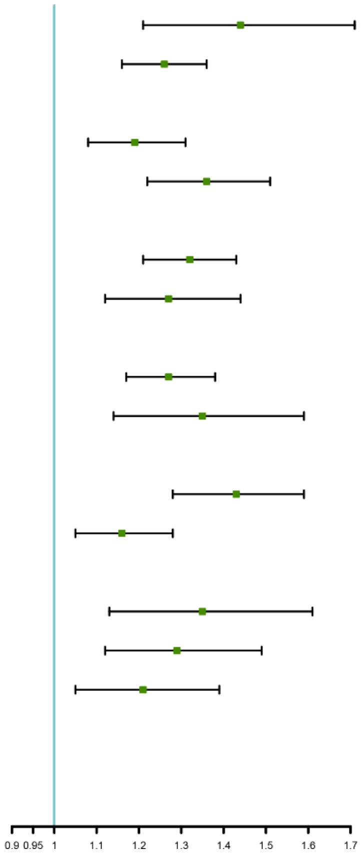

Supplement: Supplementary file 1 [file DataSheet1.pdf]
